# GP73-mediated secretion of PKM2 and GP73 promotes angiogenesis and M2-like macrophage polarization in hepatocellular carcinoma

**DOI:** 10.1038/s41419-025-07391-9

**Published:** 2025-02-05

**Authors:** Shujie Wang, Tongjia Zhang, Yue Zhou, Zitao Jiao, Kejia Lu, Xinyi Liu, Wei Jiang, Zhe Yang, Hui Li, Xiaowei Zhang

**Affiliations:** 1 Department of Biochemistry and Molecular Biology, School of Basic Medical Sciences, Beijing Key Laboratory of Protein Posttranslational Modifications and Cell Function, Peking University Health Science Center, Beijing, 100191 China; 2 Department of Pathology, The First Affiliated Hospital of Kunming Medical University, Kunming, 650032 Yunnan China

**Keywords:** Diagnostic markers, Cancer microenvironment, Tumour biomarkers

## Abstract

Hepatocellular carcinoma (HCC) is one of the most common malignant tumors. Abnormally high expression of Golgi protein 73 (GP73) and pyruvate kinase M2 (PKM2) is intimately associated with HCC progression. However, as secreted proteins, the role of their extracellular secretions in HCC progression remains unclear. Here, we demonstrated that the expression of extracellular GP73 was positively correlated with extracellular PKM2. GP73 interacted with PKM2 to promote SUMO1 modification of PKM2, which in turn enhanced the interaction of GP73 and PKM2. This process continuously promoted the transfer of PKM2 from the cytoplasm to the membrane in HCC cells, and finally secretion. Extracellular PKM2 and GP73 synergistically promoted angiogenesis and polarization of M2-type macrophages, thereby leading to malignant progression and sorafenib resistance in HCC. Sorafenib combined with shikonin, a specific inhibitor of PKM2, has a strong anti-tumor effect. This study reveals the role of GP73 in enhancing PKM2 and GP73 secretion in promoting HCC progression, providing a theoretical basis and drug targets for HCC therapy.

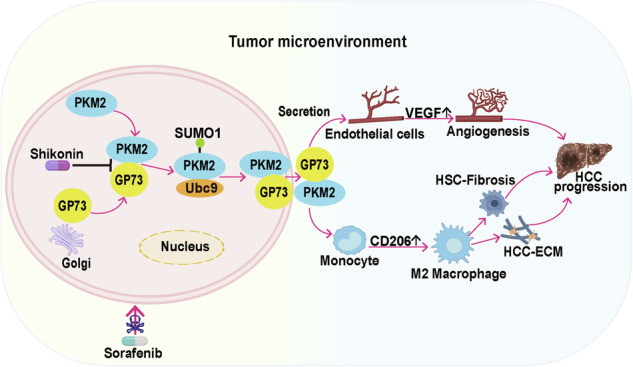

## Introduction

Liver cancer is one of the most common malignant tumors in clinical practice. According to statistics, the global liver cancer incidence and mortality rate in 2022 are ranked sixth and third, respectively, with 90% of primary liver cancers being hepatocellular carcinoma (HCC) and the 5-year survival rate being 18% [1, 2]. The main pathogenic factors of HCC are hepatitis B virus (HBV) and hepatitis C virus (HCV) infection, aflatoxin, alcohol consumption, smoking, and non-obese non-alcoholic fatty liver disease (NAFLD) [3, 4]. Therefore, a treatment strategy for liver transplantation can be adopted for early HCC. However, as HCC is generally found in the middle and late stages of the disease, there are currently no effective treatments. Sorafenib is a first-line drug in the clinical treatment of HCC, but it still has the problems of high mortality and poor prognosis due to drug resistance. Therefore, further screening of markers related to HCC occurrence and development, particularly those that can be easily measured, such as serum biomarkers, and elucidating their roles in HCC development are of great clinical value for diagnosing and treating HCC.

AFP is a widely used marker for HCC diagnosis, but it has low sensitivity, and many HCC patients have no abnormal AFP levels, especially in the early stage [5]. Therefore, the application of AFP is limited. Accumulating data show that Golgi protein 73 (GP73) is highly expressed in HCC cells and is expected to be used as a serum marker for HCC diagnosis [6]. GP73, also known as GOLM1 or GOLPH2, is a type II Golgi-localized integrated membrane protein mainly expressed in epithelial cells and at low levels in normal hepatocytes. Serum GP73 levels increase with the malignant development of liver diseases, such as hepatitis, cirrhosis, and HCC, and its sensitivity and specificity are higher than those of AFP [6]. GP73 can bind to MMP2 or MMP7, leading to their transport and secretion, ultimately promoting HCC cell metastasis [7, 8]. Our previous study showed that GP73 interacts with AFP, mediating both AFP and its own extracellular secretion. The secreted AFP and GP73 synergistically promoted the proliferation and metastasis of HCC cells [9]. A recent study has shown that GP73 can promote the malignant progression of HCC by regulating the tumor microenvironment (TME), but its regulatory mechanism needs further explored [10].

TME plays an important role in the proliferation and migration of HCC cells. In addition, cancer-associated fibroblasts (CAF) and tumor-associated macrophages (TAM) in the TME secrete a variety of cytokines that promote extracellular matrix (ECM) remodeling and angiogenesis, leading to sorafenib resistance [11]. Thus, the secreted proteins that regulate the TME have received much attention as potential molecular targets for cancer therapy [12]. It has been reported that pyruvate kinase M2 (PKM2) can be secreted extracellularly to promote HCC progression by inducing macrophage differentiation and remodeling the TME [13]. PKM2 is mainly present in the cytoplasm and is a key enzyme in the last step of glycolysis, specifically catalyzing phosphoenolpyruvate (PEP) to produce pyruvate [14]. In addition, PKM2 acts as a protein kinase in the nucleus to activate the expression of oncogenic genes such as *VEGF*, *c-myc*, and *MEK5* [15, 16]. However, recent studies have shown that elevated serum PKM2 levels are closely related to malignant progression and drug resistance in tumors. Serum PKM2 may be a potential biomarker for improving the diagnosis and predicting prognosis of patients with HCC [13, 17]. However, the extracellular secretion of PKM2 and its regulatory effect on TME remain to be elucidated.

Here, we demonstrate that PKM2 secretion is dependent on GP73. GP73-mediated PKM2 and its own extracellular secretion synergistically promote angiogenesis and polarization of M2-type macrophages, which in turn promotes proliferation and metastasis of HCC cells and enhances resistance to sorafenib. Shikonin, an inhibitor of PKM2, enhances sorafenib sensitivity by inhibiting GP73-mediated PKM2 secretion. Therefore, the combination of sorafenib and shikonin may have a stronger anti-tumor effect. Our study provides two potential biomarkers and drug targets for HCC therapy.

## Materials and methods

### Cell culture, transfection, lentivirus infection, and CRISPR/Cas9

HepG2, Huh7 (human HCC cell line), and HEK293T cells were cultured in Dulbecco’s modified Eagle’s medium (DMEM) supplemented with 10% fetal bovine serum (FBS) at 37 °C in 5% CO_2_. Transfection of plasmid cDNA was performed using the TurboFect transfection reagent (Thermo Scientific, R0533) according to the manufacturer’s protocol. The cells were collected after 48 h transfection to confirm transfection efficiency. PLVX-IRES-GP73 was transfected into HepG2 and Huh7 cells to obtain stable transfection cell lines. The lentivirus plasmid pLL3.7-shPKM2-1 expressed shRNA targeting PKM2 mRNA (5’-GCCCGAGGCTTCTTCAAGAAG-3’), pLL3.7-shPKM2-2 expressed shRNA targeting PKM2 mRNA (5’-GGTGACAGCTTCCTTTCCTGTCG -3’).

The sgRNA was designed by Zhang Feng’s lab and targeted GP73 5’-TTGGCAGTAAATCGTGTAGGCC-3,’ which was inserted into the CRISPR vector Cas9-puro-PX459. HepG2 and Huh7 cells were transfected with the expression plasmid and screened using puromycin. After three weeks, the knockout efficiency was detected.

### Real-time PCR

Cell RNA was extracted using the RNAsimple Total RNA Kit (Tiangen). cDNA was synthesized using the RevertAid First Strand cDNA Synthesis Kit (Thermo Scientific). The mRNA expression level was measured by real-time PCR using Maxima SYBR Green qPCR Master Mix (Thermo Scientific). *GAPDH* was used to normalize mRNA expression. Primer sequences are listed in Supplementary Table 1.

### Western blotting

Cells were lysed in RIPA lysis buffer (Thermo Scientific) containing a protease inhibitor cocktail (Sigma), and protein concentrations were measured using a BCA protein analysis kit (Pierce). Overall, 30–50 μg of protein was separated by SDS-PAGE and transferred to nitrocellulose membranes (Pall). Infrared fluorescence images were obtained using an Odyssey infrared imaging system (LI-COR Biosciences, Lincoln, NE, USA). The antibodies used are listed in Supplementary Table 2.

### PM protein extraction

Cell plasma membrane (PM) proteins were extracted using the Membrane and Cytosol Protein Extraction Kit (Beyotime) according to the manufacturer’s protocol. Extracted PM and cytosolic proteins were further analyzed by western blotting.

### Immunoprecipitation (IP)

Cells were harvested and lysed in IP lysis buffer (25 mM Tris-HCl (pH 7.4), 150 mM NaCl, 1% NonidetP-40, 1 mM EDTA, and 5% glycerol) containing a protease inhibitor cocktail for 1 h at 4 °C. The lysates were then centrifuged and incubated at 4 °C overnight with antibodies and protein A-Sepharose (GE Healthcare). The beads were washed thrice with IP lysis buffer and boiled in 4X SDS loading buffer at 95 °C for 10 min. The antigen-antibody complexes were subjected to western blotting with the indicated antibodies.

### TCGA data analysis

To analyze the relative mRNA expression level of GP73 in normal tissues and hepatocellular carcinoma (HCC), we used The Cancer Genome Atlas (TCGA) database with an online tool (http://gepia2.cancer-pku.cn/). To analyze the protein expression level of GP73 in normal tissues and HCC, we used the Clinical Proteomic Tumor Analysis Consortium (CPTAC) database using an online tool (http://ualcan.path.uab.edu/index.html). To evaluate the correlation between GP73 protein levels and the overall survival of HCC patients, we used The Cancer Genome Atlas (TCGA) database with an online tool (http://ualcan.path.uab.edu/index.html).

### Enzyme-linked immunosorbent assay (ELISA)

To quantitate PKM2 from HCC cell culture supernatant, a Human PKM2 ELISA Kit was used (JLC4721, BNCC) based on the manufacturer’s protocol.

### Cell migration and invasion assay

The migration and invasion assays were conducted in Transwell chambers containing 8 μm core filters (Corning). For the cell migration assay, 5 × 10^4^ cells were added to each upper chamber with 100 μl serum-free medium, and the lower chambers were filled with DMEM supplemented with 10% FBS. For the cell invasion assay, pre-diluted matrigel was added to the Transwell inserts. The following experimental procedure was the same as that for the migration assay. After 24 h of incubation, metastatic cells were fixed with 4% PFA for 30 min and stained with 0.1% crystal violet. The samples were then rinsed with deionized water to remove excess dye solution. The metastatic cells were observed under a microscope (Leica), and the number of cells in at least three fields of view was counted using the Photoshop software.

### Wound healing assay

The cells were plated in 3.5 cm dishes to almost total confluence in 24 h. Cells were scratched with the tip of a sterile microtubule and washed twice with PBS. The scratches were captured at 0 and 24 h using a microscope (Leica) and analyzed using ImageJ software. Cell repair ratio (%) = (area 0 h – area 24 h)/ area 0 h × 100.

### Cell adherence assay

The cells were seeded in 96-well plates and treated with purified proteins for 24 h. The cells were washed twice with PBS, fixed with 4% PFA, and stained with 0.1% crystal violet in 20% methanol at room temperature. After 15 min, the cells were rinsed with PBS to remove excess dye solution, crystal violet was eluted from the cells with 20% acetic acid, and the absorbance at 595 nm was recorded.

### Flow cytometry

To measure the expression of macrophage markers, THP-1 cells were cultured for 48 h with purified protein or conditioned medium (CM). After incubation, the cells were labeled with appropriate concentrations of conjugated antibodies according to the manufacturer’s instructions, and then measured by flow cytometry.

### In vivo SUMO1 conjugation assay

Huh7 cells were transfected with the indicated plasmids for the individual experiments. Forty-eight hours after transfection, the cells were collected and lysed in 1% immunoprecipitation (IP) lysis buffer containing a protease inhibitor cocktail, 10 mM iodoacetamide, and 20 mM N-ethylmaleimide. The lysates were centrifuged and incubated overnight at 4 °C with an anti-HA antibody and Protein A-Sepharose (GE Healthcare). The beads were collected and washed three times with IP lysis buffer. The antigen-antibody complexes were recovered, boiled in 4X SDS loading buffer, and subsequently subjected to western blotting with the indicated antibodies.

### Immunofluorescence (IF) assay

After washing three times, the cells were fixed with 4% paraformaldehyde (PFA) for 15 min and permeabilized (with 0.2% Triton X-100 in PBS) for 10 min at room temperature. After washing three times, the cells were blocked with 1% bovine serum albumin (BSA) in PBS for 1 h at room temperature and incubated overnight with primary antibodies at 4 °C. On the second day, the cells were incubated with the secondary antibodies. Nuclei were stained with DAPI at a concentration of 1 μg/ml for 2 min. Images were captured using a fluorescence microscope (Zeiss).

### GST pull-down assay

The control GST and other GST-tagged proteins were expressed in *Escherichia coli* strain BL21 (DE3). Bacterial lysates were prepared ultrasonically in ice-cold binding buffer (PBS) and incubated overnight in a 4 °C shaker with glutathione-Sepharose beads (GE Healthcare). The next day, His-tagged proteins were added to each sample tube and incubated for 4 h at 4 °C. The beads were washed three times, boiled in SDS-PAGE loading buffer, and then analyzed by western blotting with specified antibodies.

### Patient samples and immunohistochemistry (IHC) staining

HCC tissue samples were obtained from the Department of Pathology, First Affiliated Hospital of Kunming Medical University. We obtained verbal informed consent from all the participants. The tissue samples were dewaxed, antigen retrieved, and incubated with specific antibodies. The next day, the samples were treated with the specific secondary antibodies. The sections were exposed to DAB for 3 min and rinsed with deionized water to terminate the DAB reaction.

### Tube formation assay

Matrigel (50 μl) was added to pre-chilled 96-well plates and incubated at 37 °C for 30 min. HUVEC under different conditions were then added to the surface of the Matrigel at 3 × 10^4^ cells/well at 37 °C for 4–6 h. The formed tubes were imaged using a light microscope (Leica), and ImageJ software was used for the statistical analysis of tube formation.

### In vivo anticancer therapy

All animal experiments and facilities were approved by the Committee for Ethics of Animal Experiments and were conducted under the Guidelines for Animal Experiments, Peking University Health Science Center. 5 × 10^6^ HepG2 cells were subcutaneously injected into 5-week-old male BALB/c nude mice. When the tumor was established, the mice were randomly divided into four groups of five mice each. The mice were then treated with vehicle, sorafenib (30 mg/kg/day), shikonin (5 mg/kg/day), and sorafenib (30 mg/kg/day) with shikonin (5 mg/kg/day). On the 31st day, the mice were sacrificed and the tumors were measured and weighed.

### In vivo Matrigel plug assay

Six weeks old male BALB/c nude mice were randomly divided into four groups of three mice each. Pretreated HUVEC (200 μL, 5 × 10^6^), 300 μl Matrigel, and the specified purified proteins were mixed. The Matrigel plug was then injected subcutaneously into the right dorsal region of each nude mouse. On the 10th day, the Matrigel plugs were collected, and the blood vessel density on the Matrigel plug capsule was calculated. The samples were fixed with 10% formaldehyde, embedded in paraffin, and stained using immunohistochemistry and hematoxylin and eosin (H&E). Three random microscopic fields per sample were considered for analysis of microvessel density (MVD) inside the Matrigel plugs.

### Statistical analysis

SPSS 17.0 software and GraphPad Prism 9 were used for statistical analysis. Data are expressed as mean ± standard deviation (SD) of at least three independent experiments. Student’s *t* test was used for calculating the differences between two groups. One-way ANOVA was used to evaluate differences among multiple groups. *p* <0.05 was considered statistically significant. *p <0.05, ** *p* <0.01, *** *p* <0.001; n.s., no significance.

## Results

### GP73 promotes PKM2 secretion

**Fig. 1:**
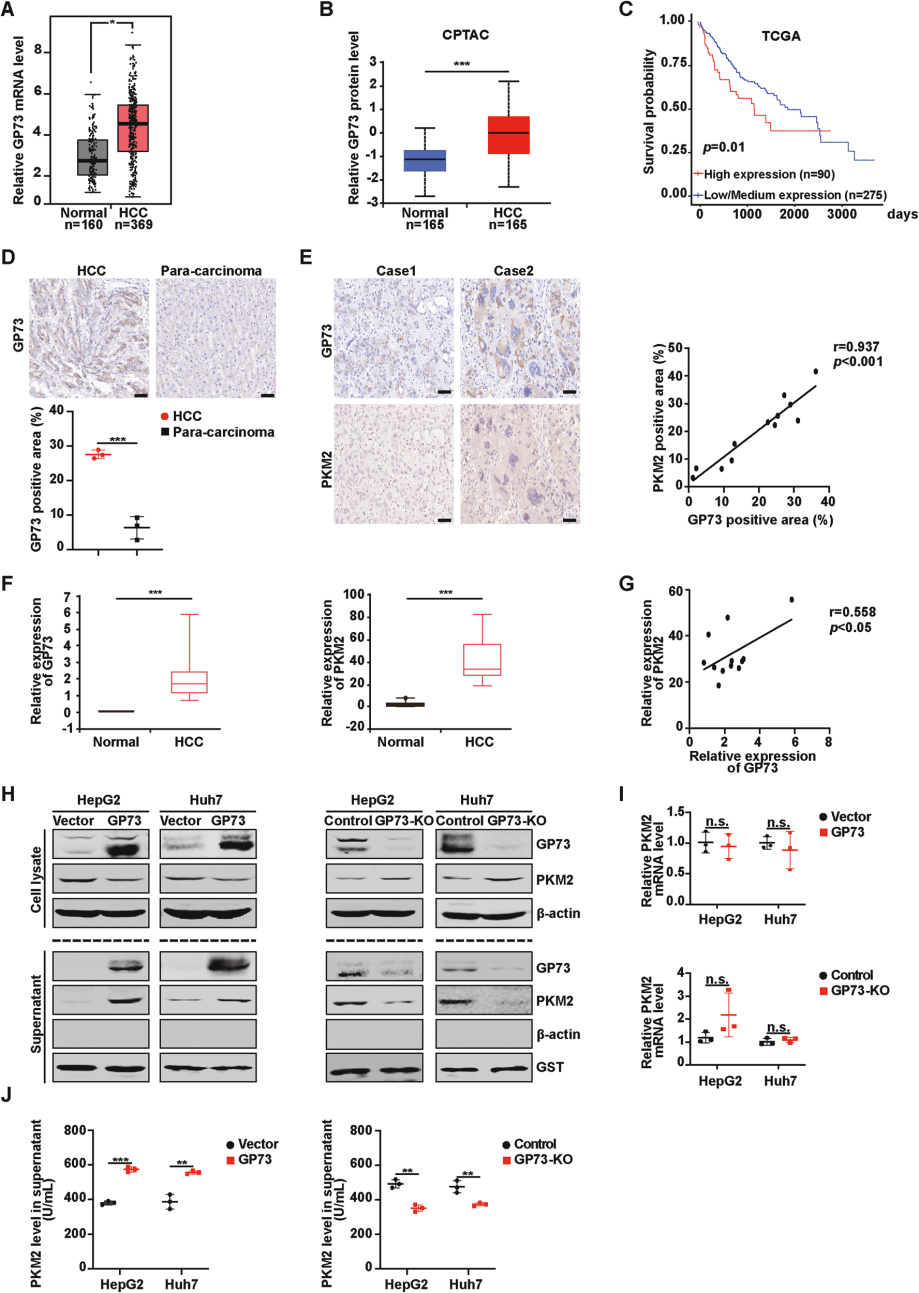
GP73 promotes PKM2 secretion. **A** Relative mRNA level of GP73 in normal and HCC tissues from TCGA database (http://gepia2.cancer-pku.cn/). **B** Relative protein levels of GP73 in normal and HCC tissues obtained from the CPTAC database (http://ualcan.path.uab.edu/index.html). **C** Kaplan-Meier plots of overall survival in two groups with high or low/medium levels of GP73 from TCGA database (http://ualcan.path.uab.edu/index.html). **D** Representative IHC images of GP73 expression in cancerous and adjacent tissues of patients with HCC and statistical evaluation of the GP73 staining area. Scale bar, 50 μm. **E** Representative IHC images of GP73 and PKM2 protein levels in serial sections of patients with HCC (*n* = 12) (left panel). Pearson’s correlation analysis of GP73 and PKM2 protein levels in HCC tissues (right panel). Scale bar, 50 μm. **F** The expression of GP73 and PKM2 in the blood of HCC patients was evaluated using the BBCancer Atlas (http://bbcancer.renlab.org/). **G** Pearson’s correlation analysis of GP73 and PKM2 expression in the blood of patients with HCC. **H** GP73 overexpression decreases the protein level of intracellular PKM2, increases the expression of extracellular PKM2, and GP73 KO increases the protein level of intracellular PKM2, and decreases the expression of extracellular PKM2. The protein levels of GP73 and PKM2 in the cell lysate and supernatant after GP73 overexpression (left panel) or KO (right panel) in HCC cells were determined by western blotting. β-actin was used as an intracellular loading control, and GST was used as an extracellular loading control. **I** GP73 does not affect the mRNA level of PKM2. The mRNA levels of PKM2 in HCC cells after GP73 overexpression (top) or KO (bottom) were measured by Real-time PCR. *n* = 3 per each group. **J** GP73 overexpression increases the secretion of PKM2 and GP73 KO decreases the secretion of PKM2. PKM2 levels in the HCC cell supernatant after GP73 overexpression (left panel) or KO (right panel) were measured by ELISA. *n* = 3 per each group. Error bars represent the mean ± SD. *p <0.05, ** *p* <0.01, *** *p* <0.001; n.s., no significance.

We analyzed the expression of GP73 in patients with HCC using The Cancer Genome Atlas (TCGA) and Clinical Proteomic Tumor Analysis Consortium (CPTAC) databases and found that the mRNA and protein expression levels of GP73 in cancer tissues were higher than those in normal tissues (Fig. 1A, B). In addition, patients with HCC with higher GP73 expression had lower survival rates (Fig. 1C). Similarly, we collected samples from HCC patients to evaluate the expression levels of GP73 in HCC and para-carcinoma by immunohistochemistry (IHC) staining. We observed significantly high GP73 expression in HCC tissues (Fig. 1D). Overall, these findings suggest that GP73 is highly expressed in HCC, and HCC patients with abnormally high GP73 expression have a lower overall survival.

Clinical studies have shown that serum levels of both PKM2 and GP73 are elevated in patients with HCC, but the specific mechanism of PKM2 secretion remains unclear [13]. Our previous study showed that GP73 can mediate the secretion of AFP, leading to the proliferation and migration of HCC cells [9]. Therefore, we hypothesized that GP73 could promote the secretion of PKM2, thereby remodeling the TME. First, we evaluated the protein expression levels of GP73 and PKM2 in pathological sections of HCC patients using IHC. We observed a significant positive correlation between GP73 and PKM2 protein levels in the HCC pathological sections (Fig. 1E). Second, we analyzed the data of blood samples from patients with HCC using a public database to evaluate the relative expression of PKM2 and GP73. As shown in Fig. 1F, the GP73 level in the blood of patients with HCC was significantly higher than that in normal controls. The PKM2 level was the same as that of GP73. There was a positive correlation between GP73 and PKM2 in the HCC blood (Fig. 1G). To further confirm whether GP73 can promote the secretion of PKM2, we overexpressed GP73 in HepG2 and Huh7 cells or knocked out GP73 using the GRISPR/Cas9 technique and detected the levels of PKM2 and GP73 by western blotting. As shown in Fig. 1H, GP73 overexpression decreased the level of intracellular PKM2 and increased the secretion of PKM2 and GP73 in the supernatant. GP73 knockout (KO) increased intracellular PKM2 levels and reduced secreted PKM2 and GP73 protein levels in the supernatant. However, GP73 did not affect PKM2 mRNA levels in HepG2 and Huh7 cells (Fig. 1I), indicating that the regulation of PKM2 by GP73 occurs at the protein level. PKM2 did not affect GP73 secretion (Supplementary Fig. 1A-C). Next, HepG2 and Huh7 cell culture supernatants were collected and PKM2 levels were determined by ELISA. The data showed that PKM2 levels increased in the supernatant of cells that overexpressed GP73, while PKM2 levels decreased in the supernatant of cells that knocked out GP73, consistent with previous results (Fig. 1J).

In summary, the protein levels of GP73 and PKM2 increased both inside and outside HCC cells, and the two proteins are positively correlated. GP73 promotes the extracellular secretion of PKM2.

### GP73-mediated PKM2 secretion depends on the interaction of GP73 with PKM2

**Fig. 2:**
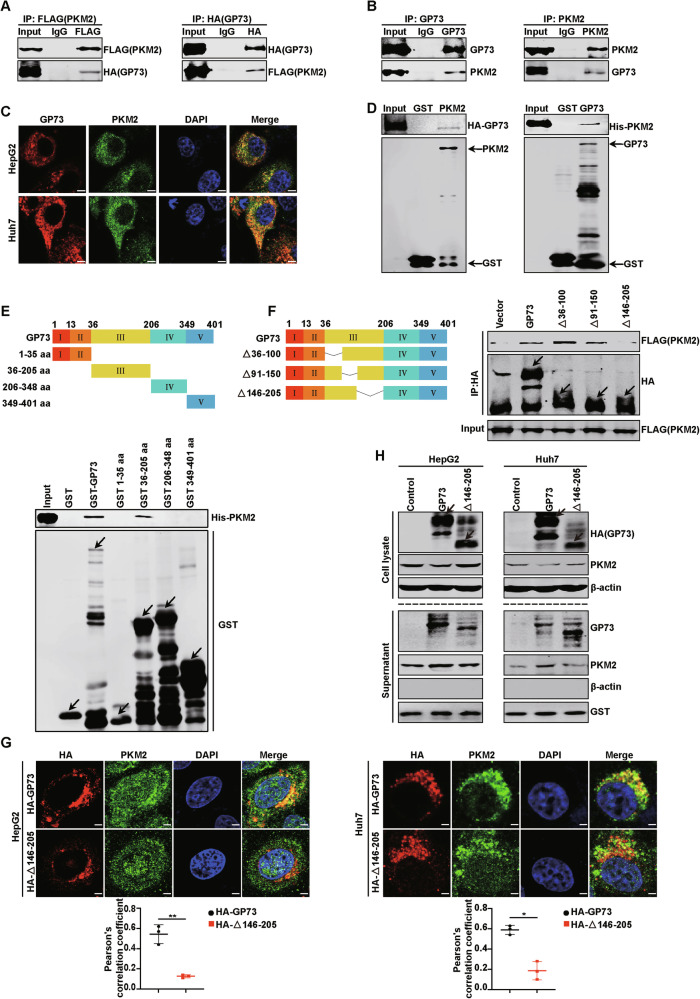
GP73-mediated PKM2 secretion depends on the interaction of GP73 with PKM2. **A**–**C** GP73 interacts with PKM2 in vivo. **A** The interaction between exogenous GP73 and PKM2 was confirmed in HEK293T cells. HEK293T cells co-transfected with FLAG-PKM2 and HA-GP73 were lysed with IP lysis buffer and Co-IP assay was carried out by using anti-FLAG/HA antibody and followed by western blotting with anti-FLAG/HA antibody. **B** The interaction between endogenous GP73 and PKM2 was confirmed in HepG2 cells. HepG2 cells were lysed with IP lysis buffer and subjected to immunoprecipitation with anti-GP73 or anti-PKM2 antibody followed by western blotting with anti-GP73 or anti-PKM2 antibody. **C** IF images reveal the colocalization of GP73 and PKM2 in the cytoplasm. HCC cells were stained with GP73 antibody (red) and PKM2 antibody (green). DAPI (blue), nucleus. Scale bar, 5 μm. **D** GP73 interacts with PKM2 in vitro. GST pull-down assay was performed by using HA-GP73/His-PKM2 with GST or GST-PKM2/GST-GP73 followed by western blotting with anti-HA/His antibody. **E** Schematic diagram of the truncated regions of GP73 and the interaction of GP73 with PKM2. PKM2 interacts with domain III of GP73. GST pull-down assay was performed by using His-PKM2 and GST or GST-tagged various domains of GP73 protein and followed by western blotting with anti-His antibody. Arrows indicate the location of target proteins. **F** Schematic diagram of the GP73 functional region and the interactions between PKM2 and deletion mutant GP73. PKM2 interacts with 146-205 aa in domain III of GP73. HEK293T cells co-transfected with FLAG-PKM2 and HA-tagged GP73, Δ36-100, Δ91-150, or Δ146-205 were lysed by IP lysis buffer and subjected to immunoprecipitation with anti-FLAG/HA antibody followed by western blotting with anti-FLAG/HA antibody. Arrows indicate the location of the target proteins. Δ represents the deletion of amino acid residues. **G** Top, IF images show that HCC cells co-transfected with HA-Δ146-205 and PKM2 are less co-localized than those co-transfected with HA-GP73 and PKM2. HCC cells co-transfected with PKM2 and HA-tagged GP73 or Δ146-205 were stained with HA antibody (red) and PKM2 antibody (green). DAPI (blue), nucleus. Scale bar, 2 μm. Bottom, Pearson’s correlation analysis of the correlation between PKM2 and GP73 or Δ146-205 in HCC cells. **H** Deletion of 146-205 aa in domain III of GP73 reduces PKM2 secretion. The protein levels of GP73 and PKM2 in cell lysate and supernatant were measured by western blotting in HCC cells transfected with vector, GP73 or Δ146-205 plasmid. β-actin was used as an intracellular loading control, and GST as an extracellular loading control. Arrows indicate the location of the target proteins. Δ represents the deletion of amino acid residues. Error bars represent mean ± SD (*n* = 3). *p <0.05**, *p* <0.01.

Next, the specific mechanism by which GP73 promoted PKM2 secretion was explored. We first investigated whether GP73 interacted with PKM2. FLAG-PKM2 and HA-GP73 expression plasmids were co-transfected into HEK293T cells, and the cells were collected for the Co-IP assay. As shown in Fig. 2A, exogenous GP73 interacted with exogenous PKM2 in vivo. Next, the interaction of endogenous GP73 with PKM2 was assessed using a Co-IP assay. Similar to exogenous proteins, endogenous GP73 also bound to endogenous PKM2 (Fig. 2B). IF images also indicated that GP73 colocalized with PKM2 in the cytoplasm (Fig. 2C). In addition, a GST pull-down assay was performed to determine whether GP73 and PKM2 directly interacted. There was a direct interaction between GP73 and PKM2 in vitro (Fig. 2D). As noted above, GP73 physically interacts with PKM2 in vivo and in vitro. To explore whether the interaction of GP73 with PKM2 affects the secretion of PKM2, we used a set of GST-labeled GP73-deletion mutants in the GST pull-down experiment to identify the domains required for GP73 interaction. The results showed that domain III (amino acid residues 36-205) of GP73 directly bound to PKM2, whereas the other domains of GP73 did not (Fig. 2E). To identify a more specific region of GP73 combined with PKM2, we constructed three deletion mutants of domain III and performed Co-IP assays. As shown in Fig. 2F, Δ146-205 (deletion of amino acid residues 146-205) could not bind to PKM2, whereas the other two mutants did. These results illustrated that the region spanning amino acid residues 146-205 is necessary for the combination of GP73 with PKM2. Similarly, IF images showed that HCC cells co-transfected with HA-Δ146-205 and PKM2 were less co-localized than those co-transfected with HA-GP73 and PKM2 (Fig. 2G). In this assay, HA-GP73 and HA-Δ146-205 were stained red with an anti-HA antibody, and PKM2 was stained green with an anti-PKM2 antibody. The IF merged images shown in yellow represent their combination. Next, HepG2 and Huh7 cells were respectively transfected with the vector, wild-type GP73, and the GP73 truncated mutant Δ146-205. Western blotting was used to measure intracellular and supernatant PKM2 protein levels. Compared to the vector, GP73 overexpression reduced the protein levels of intracellular PKM2 and increased the levels of extracellular PKM2, whereas Δ146-205 did not affect the protein expression of intracellular and extracellular PKM2 (Fig. 2H).

Overall, these results illustrated that GP73-mediated PKM2 secretion is dependent on the interaction of GP73 with PKM2 and requires amino acid residues 146-205 of GP73 specifically.

### GP73 promotes PKM2 secretion by enhancing Ubc9-mediated SUMO1 modification of PKM2

**Fig. 3:**
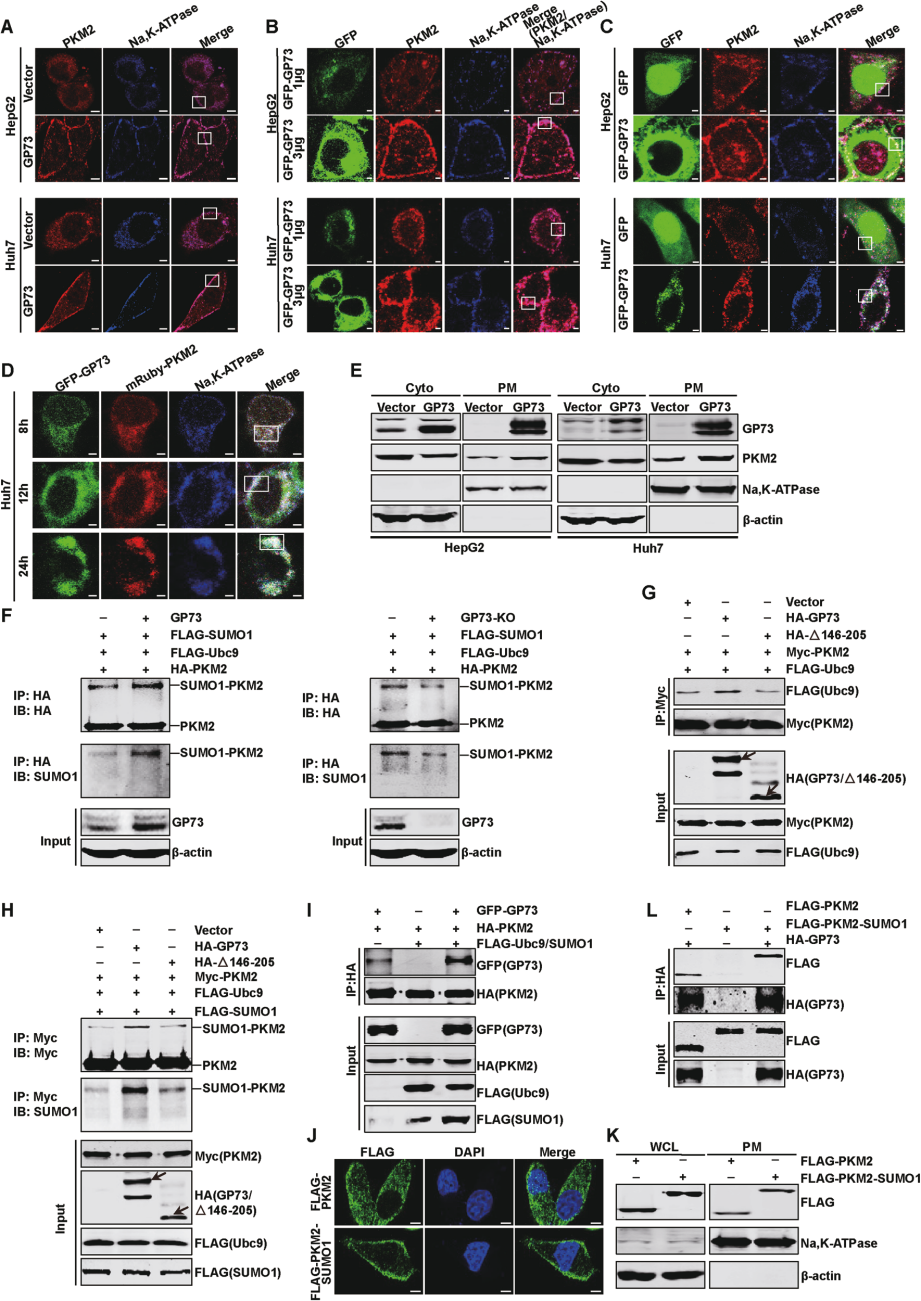
GP73 promotes PKM2 secretion by enhancing Ubc9-mediated SUMO1 modification of PKM2. **A**, **B** GP73 increases the PM location of PKM2. **A** IF merged images reveal more PM location of PKM2 in HCC cells transfected with GP73 compared to vector. Na, K-ATPase was used as a plasma membrane marker. PKM2 (red), Na, K-ATPase (blue). Scale bar, 5 μm. The white rectangles represent the location of PKM2 in the PM. **B** IF merged images reveal the PM location of PKM2 in HCC cells depending on the doses of GFP-GP73. Na, K-ATPase was used as a plasma membrane marker. GFP (green), PKM2 (red), Na, K-ATPase (blue). Scale bar, 2 μm. The white rectangles represent the location of PKM2 in the PM. **C** IF merged images reveal the colocalization of GP73 and PKM2 in PM of HCC cells. GFP (green), PKM2 (red), Na, K-ATPase (blue). Scale bar, 2 μm. The white rectangles represent the colocation of GP73 and PKM2 in the PM. **D** IF merged images reveal the colocalization of GP73 and PKM2 in Huh7 cells transfected with GFP-GP73 and mRuby-PKM2 at 8, 12, and 24 h. GFP-GP73 (green), mRuby-PKM2 (red), Na, K-ATPase (blue). Scale bar, 2 μm. The white rectangles represent the co-location of GP73 and PKM2. **E** GP73 overexpression increases the amount of PKM2 in the PM and decreases the amount in the cytoplasm of HCC cells as determined by western blotting. Na, K-ATPase was used as a PM protein loading control and β-actin was used as a cytoplasm loading control. **F** GP73 overexpression promotes the SUMO1 modification of PKM2 and GP73 KO attenuates the modification of PKM2. Huh7 cells co-transfected with FLAG-SUMO1, FALG-Ubc9, HA-PKM2, and control vector or GP73 were subjected to an in vivo SUMO1 conjugation assay (left panel). Huh7 control or GP73 KO cells in the right panel were transfected with FLAG-SUMO1, FALG-Ubc9, and HA-PKM2. **G** Deletion of 146-205 aa in domain III of GP73 attenuates the combination of PKM2 and Ubc9. Huh7 cells co-transfected with indicated plasmids were lysed with IP lysis buffer, and Co-IP assay was carried out. The arrows indicate the location of the target proteins. **H** Deletion of 146-205 aa in domain III of GP73 attenuates the SUMO1 modification of PKM2. Huh7 cells co-transfected with indicated plasmids were subjected to an in vivo SUMO1 conjugation assay. The arrows indicate the location of the target proteins. **I** The SUMO1 modification of PKM2 promotes the combination of PKM2 and GP73. Huh7 cells co-transfected with indicated plasmids were lysed with IP lysis buffer, and Co-IP assay was carried out. **J**, **K** The PM location of PKM2-SUMO1 in Huh7 cells was detected by Confocal IF as in (**J**) and western blotting as in (**K**). FLAG (green), DAPI (blue), nucleus. Scale bar, 5 μm. **L** PKM2-SUMO1 promotes the combination of GP73 and PKM2. Huh7 cells co-transfected with indicated plasmids were lysed with IP lysis buffer, and an IP assay was carried out.

To further elucidate how GP73 promotes PKM2 secretion, HepG2 and Huh7 cell lines with stable GP73 expression were constructed, and IF staining was performed with anti-PKM2 antibody (red) and anti-Na, K-ATPase antibody (blue), which was the marker of the plasma membrane (PM), respectively. As shown in Fig. 3A, GP73 overexpression promoted the PM localization of PKM2 and decreased its cytoplasmic localization compared to the control group. Western blotting was also used to verify that when GP73 was overexpressed, the amount of PKM2 in the PM increased, and the amount in the cytoplasm decreased in HepG2 and Huh7 cells (Fig. 3E). When HepG2 and Huh7 cells were transfected with 3 μg GFP-GP73, PKM2 merged with Na, K-ATPase showed more PM localization than the cells transfected with 1 μg GFP-GP73 (Fig. 3B). These results suggest that GP73 promotes the movement of PKM2 from the cytoplasm to the PM and that this process depends on the amount of GP73. In addition, the GFP, PKM2, and Na, K-ATPase merged images showed the colocalization of GP73 and PKM2 in the PM (Fig. 3C). To observe the combined process of GP73 and PKM2 more intuitively, the IF assay was performed at different times. Huh7 cells were co-transfected with GFP-GP73 and mRuby-PKM2, and the cells were collected at 8, 12, and 24 h after transfection. The merged images showed that the colocalization intensity of GP73 and PKM2 on the cell membrane increased from 8 to 12 h (Fig. 3D).

Previous studies have revealed that SUMO1 modification facilitates PKM2 translocation from the cytoplasm to the PM, and high SUMO1 modification of PKM2 has been observed in HCC [13]. We confirmed that inhibiting the SUMO1 modification of PKM2 reduces its PM location and attenuates its secretion (Supplementary Fig. 2A–D). Therefore, we hypothesized that GP73 enhanced PKM2 movement from the cytoplasm to the PM is related to SUMO1 modification of PKM2. To confirm our hypothesis, GP73 overexpressing or GP73 KO cells were co-transfected with FLAG-SUMO1, FLAG-Ubc9, and HA-PKM2. As shown in Fig. 3F, GP73 overexpression enhanced the conjugation of FLAG-SUMO1 to HA-PKM2, whereas GP73 KO decreased SUMO1 modification of PKM2, indicating that GP73 promotes SUMO1 modification of PKM2.

It has been reported that the knockdown of Ubc9, the only SUMO-conjugating enzyme, can attenuate PM targeting and secretion of PKM2. In addition, Ubc9 interacts with PKM2 in HCC cells [13]. Therefore, we hypothesized that GP73-promoted SUMO1 modification of PKM2 is related to enhanced binding of Ubc9 and PKM2. We then confirmed our hypothesis, as shown in Fig. 3G, GP73 promoted the binding of Ubc9 and PKM2, and Δ146-205 lost this ability. These data suggest that the interaction between Ubc9 and PKM2 depends on that between GP73 and PKM2. To further confirm the importance of GP73 and PKM2 binding, an in vivo SUMO1 conjunction assay was performed in Huh7 cells. As shown in Fig. 3H, GP73 enhanced the conjugation of FLAG-SUMO1 to Myc-PKM2, whereas Δ146-205 had no such effect, indicating that the interaction between GP73 and PKM2 promoted SUMO1 modification of PKM2. Next, we explored the effects of SUMO1 modification of PKM2. The Co-IP assay revealed that SUMO1 modification of PKM2 promoted the binding of GP73 and PKM2 (Fig. 3I). To verify this result further, a PKM2-SUMO1 plasmid was constructed. The behavior of the SUMO-fusion protein is similar to that of the physiologically sumoylated protein [18]. PKM2-SUMO1 was mainly located in the PM (Fig. 3J, K). Furthermore, the Co-IP assay showed that PKM2-SUMO1 promoted the interaction between GP73 and PKM2 (Fig. 3L).

Overall, GP73 promotes the translocation of PKM2 from cytoplasm to PM by enhancing Ubc9-mediated SUMO1 modification of PKM2, which is related to the interaction of GP73 and PKM2, and this SUMO1 modification of PKM2 promotes the interaction of GP73 and PKM2 in turn.

### GP73-mediated secretion of PKM2 and GP73 promotes angiogenesis

**Fig. 4:**
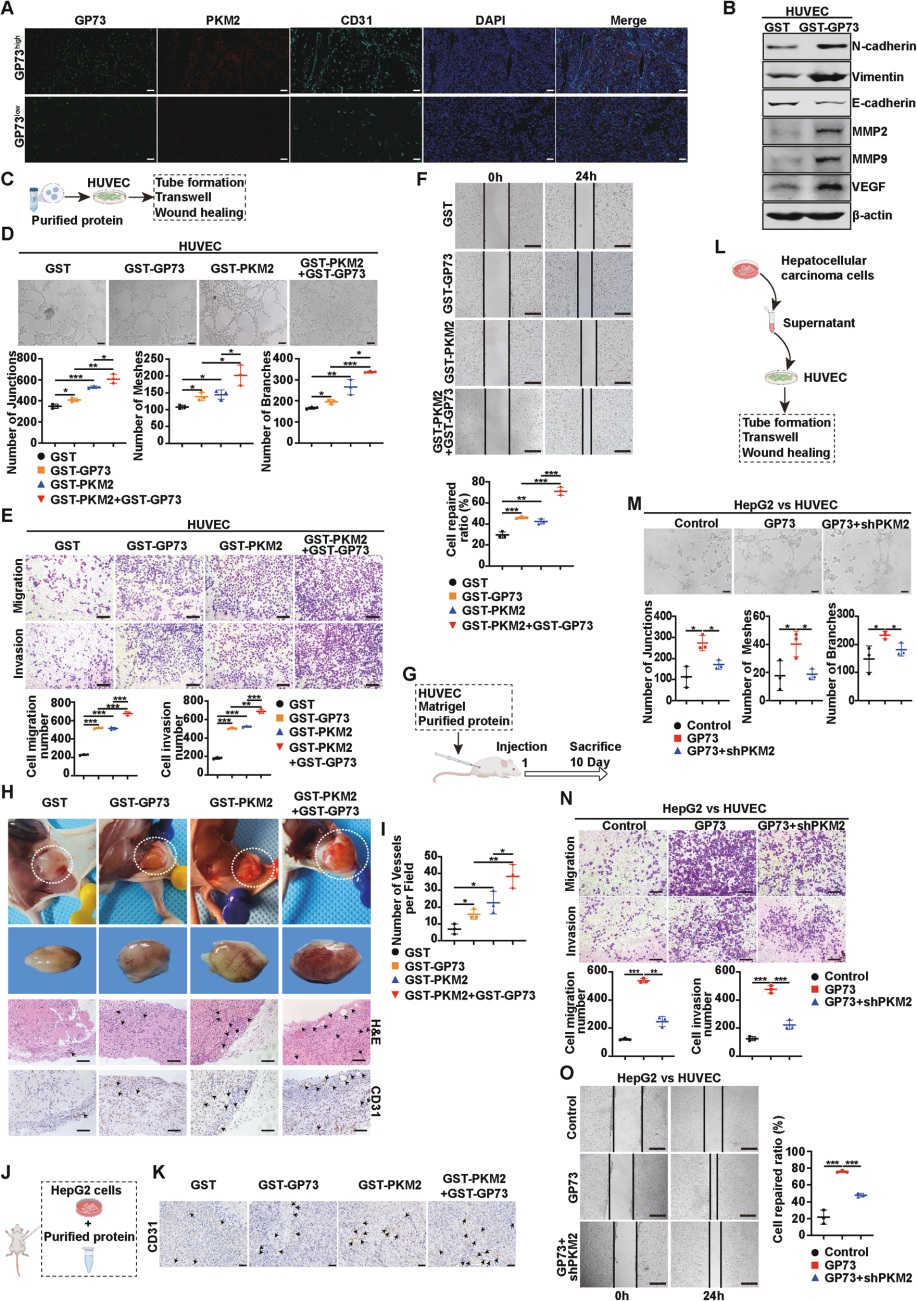
GP73-mediated secretion of PKM2 and GP73 promotes angiogenesis. **A** Representative IF staining of GP73, PKM2, and CD31 in high- and low- GP73 group of human HCC tissues. Scale bar, 100 μm. **B** Protein levels of N-cadherin, Vimentin, E-cadherin, MMP2, MMP9, and VEGF in HUVEC after incubation with GST or GST-GP73 were measured using western blotting. β-actin was used as a loading control. **C** Schematic diagram of HUVEC co-cultured with the purified protein in angiogenesis processing. **D** Top, representative images of the tube formation assay to reveal the tube-forming ability of HUVEC treated with the indicated protein. Scale bar, 100 μm. Bottom, the quantitative statistic of tube formation was measured by Image J. **E** Top, representative images of Transwell assay to show the migration and invasion potential of HUVEC treated with the indicated protein. Scale bar, 200 μm. Bottom, statistical graph of cell migration number and invasion number. **F** Top, representative images of wound healing assay to reveal the migration potential of HUVEC treated with indicated protein. Scale bar, 500 μm. Bottom, the quantitative statistic of cell repaired ratio was measured by Image J. **G** Schematic diagram of in vivo Matrigel plug assay processing. **H**, **I** Matrigel plug assay was used to evaluate the angiogenesis in vivo. **H** From top to bottom, the images of the Matrigel plug in mice; the gross form of the Matrigel plug; the Matrigel plug stained with H&E; CD31 IHC staining of the Matrigel plug. The dotted lines show the border of the Matrigel plugs, arrows represent the microvessel. Scale bar, 100 μm. **I** The statistical evaluation of the number of microvessels per field in the Matrigel plug was quantified by CD31 IHC staining. **J** Schematic diagram of purified protein during angiogenesis processing in the subcutaneous implantation tumor model. HepG2 cells and purified protein were subcutaneously injected into 5-week BALB/c nude mice. The tumors were analyzed after injection for 30 days. **K** CD31 IHC staining of the tumor. Arrows represent the microvessel. Scale bar, 50 μm. **L** Schematic diagram of HUVEC treated with HCC cells medium in angiogenesis processing. **M**–**O** PKM2 knockdown reduces GP73-induced angiogenesis. **M** Top, representative images of the tube formation assay to reveal the tube-forming ability of HUVEC treated with the indicated group supernatant of HepG2 cells. Scale bar, 100 μm. Bottom, the quantitative statistic of tube formation was measured by Image J. **N** Top, representative images of the Transwell assay to show the migration and invasion potential of HUVEC treated with the indicated group supernatant of HepG2 cells. Scale bar, 200 μm. Bottom, statistical graph of cell migration and invasion number. **O** Left, representative images of the wound healing assay to reveal the migration potential of HUVEC treated with the indicated group supernatant of HepG2 cells. Scale bar, 500 μm. Right, the quantitative statistics of cell repaired ratio was measured by Image J. Error bars represent mean ± SD (*n* = 3). *p <0.05, ** *p* <0.01, *** *p* <0.001.

The above findings showed that GP73 overexpression promoted the secretion of PKM2 and promoted its own secretion. Next, we explored the effects of secreted GP73 and PKM2 on TME. Extracellular PKM2 has been reported to promote angiogenesis [19], and we tested whether extracellular GP73 had the same function. First, we explored the correlation of GP73, PKM2 and the microvascular density marker CD31 in human primary HCC tissues. The IF staining images suggested that there were remarkably positively correlations of GP73, PKM2 and CD31 in HCC tissues (Fig. 4A). Also, we used the CPTAC database to analyze the correlation and found the same results (Supplementary Fig. 3A). Next, Purified GST or GST-GP73 protein was added to the culture medium of HUVEC. As shown in Supplementary Fig. 3B, GST-GP73 decreased the number of adherent HUVEC. Similarly, HUVEC incubated with GST-GP73 rather than GST had a lower mean fluorescence intensity (MFI) for VE-cadherin, a marker of cell adhesion (Supplementary Fig. 3C). In addition, the incubation of HUVEC with GST-GP73 led to a higher MFI of Vimentin, which represents cell migration (Supplementary Fig. 3D). Western blotting showed that incubation of HUVEC with GST-GP73 increased the protein expression levels of N-cadherin, Vimentin, MMP2, MMP9, and VEGF and decreased the protein levels of E-cadherin compared with GST (Fig. 4B). These results indicate that GST-GP73 could reduce adhesion and promote the migration of HUVEC, which lays the foundation for angiogenesis. We added the purified proteins to the medium of HUVEC individually or collectively, and then proceeded to subsequent experiments, as shown in Fig. 4C. Compared to the GST group, GST-GP73 or GST-PKM2 promoted angiogenesis. Meanwhile, the collective addition of GST-GP73 and GST-PKM2 enhanced angiogenesis in vitro more significantly than the addition of GST-GP73 or GST-PKM2 alone (Fig. 4D–F). To further determine the proangiogenic effects of GST-GP73 and GST-PKM2, we conducted in vivo experiments using nude mice, as shown in Fig. 4G. We also observed a proangiogenic phenomenon in the Matrigel plug assay in vivo (Fig. 4H, I). Consistent with the results of in vitro experiments, the GST-GP73 or GST-PKM2 group had a higher density of vessels on the Matrigel plug in the capsule, and their images of hematoxylin and eosin (H&E) staining and IHC staining of CD31 showed higher microvessel density (MVD) inside the Matrigel plug than the GST group. GST-GP73 and GST-PKM2 synergistically enhanced these effects. Besides, the IHC images of CD31 on subcutaneous implantation tumor models showed consistent results (Fig. 4J, K). These data demonstrate that GP73 and PKM2 synergistically promote angiogenesis in HCC. Emerging researches have showed that angiogenesis plays a key role in the regulation of cancer metastasis. Then we explored the effect of GP73 and PKM2 on metastasis of HCC cells using an in vivo lung metastasis model as shown in Supplementary Fig. 3I. The data showed that GST-GP73 and GST-PKM2 synergistically increased the number of lung surface metastatic nodules (Supplementary Fig. 3J, K). Besides, GST-GP73 and GST-PKM2 synergistically increased MMP2 and MMP9 expression levels in lung tumors (Supplementary Fig. 3L). Our in vivo data suggested that GP73 and PKM2 synergistically promote metastasis of HCC cells.

In addition, we examined whether decreased PKM2 secretion attenuated the pro-angiogenic effect of GP73. As shown in Fig. 4L, HUVEC were incubated with different groups of HCC media, and subsequent experiments were conducted. HepG2 and Huh7 cells were stably transfected with GP73 with or without lentiviral knockdown of PKM2. GP73 overexpression increased the secretion of its own and PKM2, while PKM2 knockdown reduced PKM2 secretion (Supplementary Fig. 3E). Next, control, GP73, and GP73+shPKM2-2 groups of HepG2 and Huh7 cells were collected and incubated with HUVEC. PKM2 knockdown reduced GP73-induced angiogenesis (Fig. 4M–O, Supplementary Fig. 3F–H), indicating that decreased secretion of PKM2 attenuates the pro-angiogenic effect of GP73, highlighting the synergistic effect of secreted GP73 and PKM2 on angiogenesis.

### GP73-mediated secretion of PKM2 and GP73 promotes monocyte-to-macrophage differentiation

**Fig. 5:**
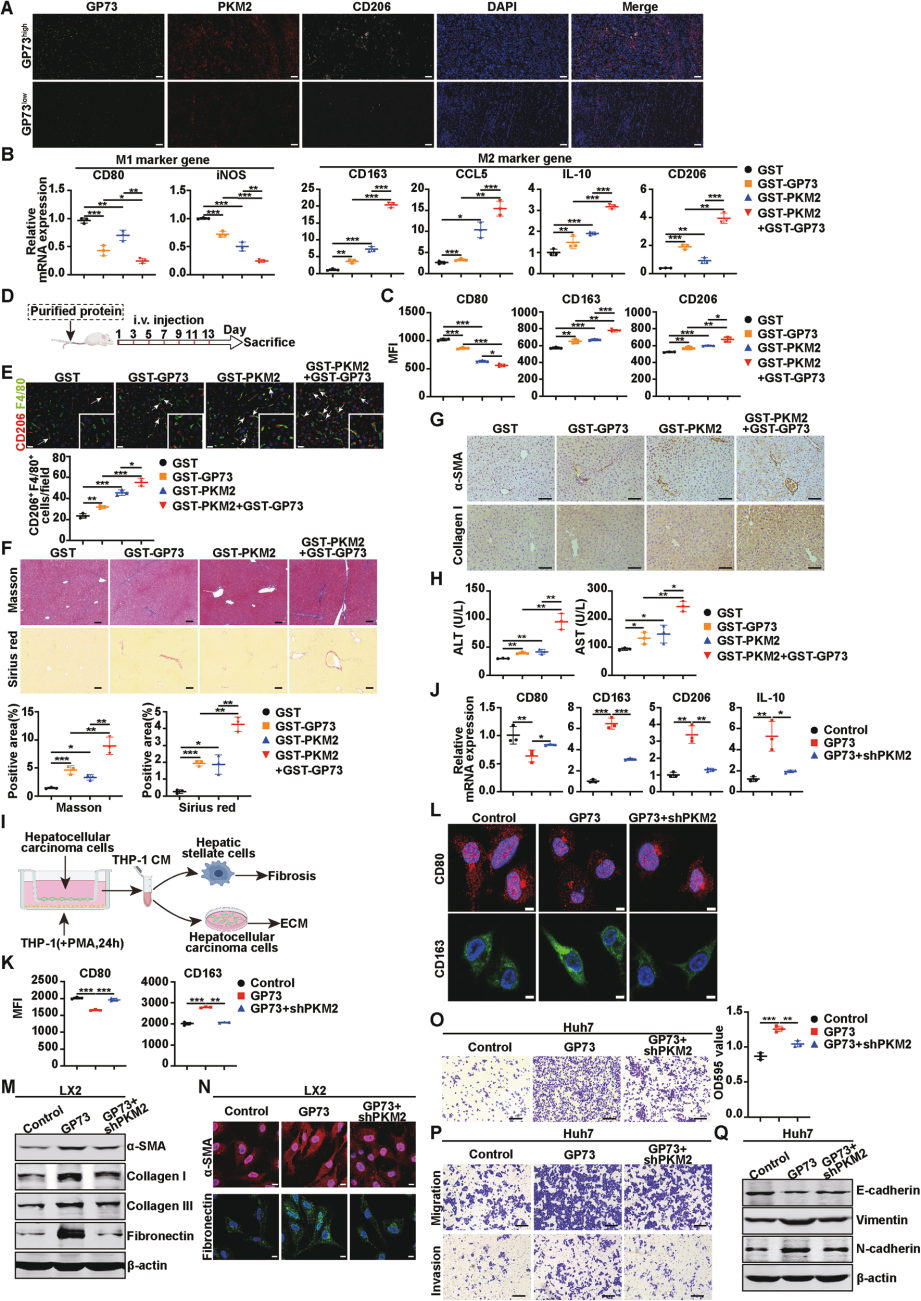
GP73-mediated secretion of PKM2 and GP73 promotes monocyte-to-macrophage differentiation. **A** Representative IF staining of GP73, PKM2, and CD206 in high- and low- GP73 group of human HCC tissues. Scale bar, 100 μm. **B** The mRNA levels of M1 and M2 macrophage marker genes were measured using Real-time PCR after THP-1 cells incubation with indicated protein. **C** Flow cytometry was used for analysis MFI of M1 and M2 marker genes after THP-1 cells incubation with the indicated purified protein. **D** Schematic diagram of experimental design to establish the animal model. **E** Top, representative IF images of CD206 (red, M2 macrophage marker) and F4/80 (green, macrophage marker) expression in mouse livers educated with the indicated protein. Scale bar, 20 μm. Bottom, the statistical graph of double-positive cells per field. The arrows indicate double-positive cells. White insets depict a higher magnification of the arrows showing the representative area. **F** Top, representative images of Masson and Sirius red staining in the livers of mice in the indicated groups. Bottom, a statistical graph of the positive area of Masson and Sirius red staining in the livers of mice in the indicated groups. Scale bar, 100 μm. **G** Representative IHC images of α-SMA (top) and Collagen I (bottom) in the livers of mice in the indicated groups. Scale bar, 100 μm. **H** Content of ALT and AST from the blood of mice in the indicated groups. **I** Schematic diagram of experimental design to establish the cell co-culture model. **J-L** PKM2 knockdown increases M1 macrophage marker expression and decreases M2 macrophage marker expression. Real-time PCR (**J**), flow cytometry (**K**), and IF assay (**L**) were conducted with THP-1 cells after incubation with the indicated culture medium of the Huh7 cells. CD80 (red), CD163 (green), DAPI (blue), nucleus. Scale bar, 5 μm. **M** The protein levels of α-SMA, Collagen I, Collagen III, and Fibronectin in LX2 cells incubated with the indicated culture medium Of Huh7 cells were measured by western blotting. β-actin was used as a loading control. **N** Representative IF images of α-SMA and Fibronectin expression in LX2 cells incubated with the indicated CM of Huh7 cells. α-SMA (red), Fibronectin (green), DAPI (blue), nucleus. Scale bar, 10 μm. **O** Left, representative cell adherence assay images of Huh7 cells incubated with the indicated CM of Huh7 cells. Right, the quantitative statistic of OD 595 value. Scale bar, 200 μm. **P** Representative images of Transwell assay of Huh7 cells incubated with the indicated CM of Huh7 cells. Scale bar, 200 μm. **Q** The protein levels of E-cadherin, Vimentin, and N-cadherin in Huh7 cells incubated with the indicated CM were measured by western blotting. β-actin was used as a loading control. Error bars represent mean ± SD (*n* = 3). *p <0.05, ** *p* <0.01, *** *p* <0.001.

Since extracellular GP73 and PKM2 promote angiogenesis, we wondered whether these two secreted proteins influence another important event in the TME, monocyte-to-macrophage differentiation. First, we explored the correlation of GP73, PKM2 and the M2 macrophage marker CD206 in human primary HCC tissues. The IF staining images suggested that there were remarkably positively correlations of GP73, PKM2 and CD206 in HCC tissues (Fig. 5A). Also, we used the CPTAC database and found the same results (Supplementary Fig. 4A). Next, we further explored the role of extracellular GP73 and PKM2 in monocyte-to-macrophage at the cellular level as shown in Supplementary Fig. 4B. THP-1 cells were incubated with PMA for 24 h, and purified proteins were added to the medium of THP-1 cells. The human monocytic cell line THP-1 was induced into macrophages by phorbol 12-myristate 13-acetate (PMA), which resulted in morphological and functional changes characterized by the transformation of monocytes to macrophages (Supplementary Fig. 4C–E) [20]. The upregulated expression of CD68 was a marker of macrophages after treatment with PMA (Supplementary Fig. 4E). Flow cytometry and Real-time PCR were used to identify the expression levels of M1 and M2 macrophage markers. Previous studies have shown that extracellular PKM2 can induce THP-1 cells to generate M2 macrophages [13, 21], whether extracellular GP73 has the same function is still unclear. We first confirmed that FITC-labeled GST-GP73 protein was phagocytosed by THP-1 cells after PMA incubation for 24 h (Supplementary Fig. 4F). The expression of the macrophage marker CD14, but not the dendritic cell (DC) marker CD83, was upregulated in THP-1 cells after incubation with GST-GP73 (Supplementary Fig. 4G), indicating that secretory GP73 induced THP-1 cells to differentiate into macrophages rather than DC. Flow cytometry results showed that the MFI of the M1 macrophage marker CD80 was decreased after incubation with GST-PKM2 or GST-GP73 individually, and significantly decreased after treatment collectively. In contrast, the MFI of the M2 macrophage markers CD163 and CD206 increased after the addition of GST-GP73 or GST-PKM2, and when these two proteins were added together, the MFI of CD163 and CD206 increased significantly (Fig. 5C, Supplementary Fig. 4H). Similarly, the results of Real-time PCR also showed that the mRNA levels of M1 macrophage marker genes *CD80* and *iNOS* were reduced after treatment with GST-GP73 or GST-PKM2, and the mRNA levels of *CD80* and *iNOS* were significantly decreased after the simultaneous addition of the two proteins, whereas the mRNA levels of M2 macrophage marker genes *CD163, CCL5, IL-10*, and *CD206* were opposite (Fig. 5B). These data suggest that extracellular GP73 collaborates with PKM2 to promote the differentiation of M0 macrophages into M2 macrophages in vitro. Next, we conducted in vivo experiments by injecting specifically purified proteins into mice through the tail vein every two days (Fig. 5D). Two weeks later, an IF assay of mouse liver tissues was conducted, consistent with the results of in vitro experiments, the IF merged images showed that the injection of GST-GP73 or GST-PKM2 increased the population of M2 macrophages (CD206^+^ F4/80^+^), and the combination of the two proteins had a synergistic effect (Fig. 5E). Next, we investigated the effect of elevated extracellular GP73 and PKM2 on liver fibrosis. Masson and Sirius Red staining showed that GST-GP73 or GST-PKM2 promoted hepatic fibrosis in mice, and the simultaneous injection of these two proteins promoted fibrosis more significantly (Fig. 5F**)**. Protein levels associated with hepatic fibrosis in mice were detected by IHC. The images showed that the expression levels of α-SMA and collagen I were increased in the liver after GST-GP73 and GST-PKM2 injection individually, and more obvious effects were observed after simultaneous injection (Fig. 5G**)**. Meanwhile, we found that after injection of GST-GP73 or GST-PKM2, the ALT and AST levels of the mice increased, and these levels increased more significantly after injection collectively (Fig. 5H**)**, suggesting that the simultaneous injection of GST-GP73 and GST-PKM2 resulted in more serious liver damage in mice. These results suggest that the purified protein GP73 can coordinate with PKM2 to promote the polarization of M2 macrophages, facilitate liver fibrosis, and lead to severe liver damage.

To further investigate the role of GP73-mediated PKM2 and GP73 secretion in the TME of HCC, HCC cells with stable expression of Vector, GP73, or GP73+shPKM2 were constructed and co-cultured with PMA-treated THP-1 cells for 24 h, and the conditioned medium (CM) was collected. The CM was then incubated with hepatic stellate cells (HSC) or HCC cells to detect its effect on fibrosis and ECM. This process is illustrated in Fig. 5I. Using Real-time PCR (Fig. 5J), flow cytometry (Fig. 5K, Supplementary Fig. 4I), and IF assays (Fig. 5L), we found that the expression level of CD80 decreased, whereas the levels of CD163, CD206, and IL-10 increased in THP-1 cells after incubation with the CM of the GP73 overexpression group of HCC cells, and PKM2 knockdown by shRNA partially reversed these effects. These findings suggest that GP73-mediated secretion of PKM2 and GP73 synergistically facilitates the polarization of M2-type macrophages in THP-1 cells, and that this effect partially depends on endogenous PKM2. CM from the three groups was collected and incubated with LX2 cells, one of the HSC. Western blotting was used to detect the expression of liver fibrosis-related proteins. The results showed that GP73 overexpression CM obviously enhanced the expression of α-SMA, collagen I, collagen III, and Fibronectin in LX2 cells. Compared with this group, co-culture with GP73+shPKM2 CM decreased the expression levels of the above proteins (Fig. 5M). This result was consistent with the effect of cell IF (Fig. 5N). These data indicate that the GP73-mediated secretion of PKM2 and GP73 synergistically facilitates hepatic fibrosis.

Moreover, the CM was collected and incubated with Huh7 cells, and the cell adhesion assay showed that GP73 overexpression promoted Huh7 cell adhesion. Compared to this group, the CM of GP73+shPKM2 reduced the adhesion ability of Huh7 cells (Fig. 5O). The migration and invasion of Huh7 cells incubated with CM from the above groups showed similar effects (Fig. 5P). The changes in the protein levels of adhesion factors also supported these results (Fig. 5Q).

In short, these data indicate that GP73-mediated secretion of PKM2 and its own can induce monocyte differentiation into M2 macrophages and subsequently promote liver fibrosis and ECM processes.

### Extracellular GP73 and PKM2 attenuate sorafenib anti-tumor efficiency

**Fig. 6:**
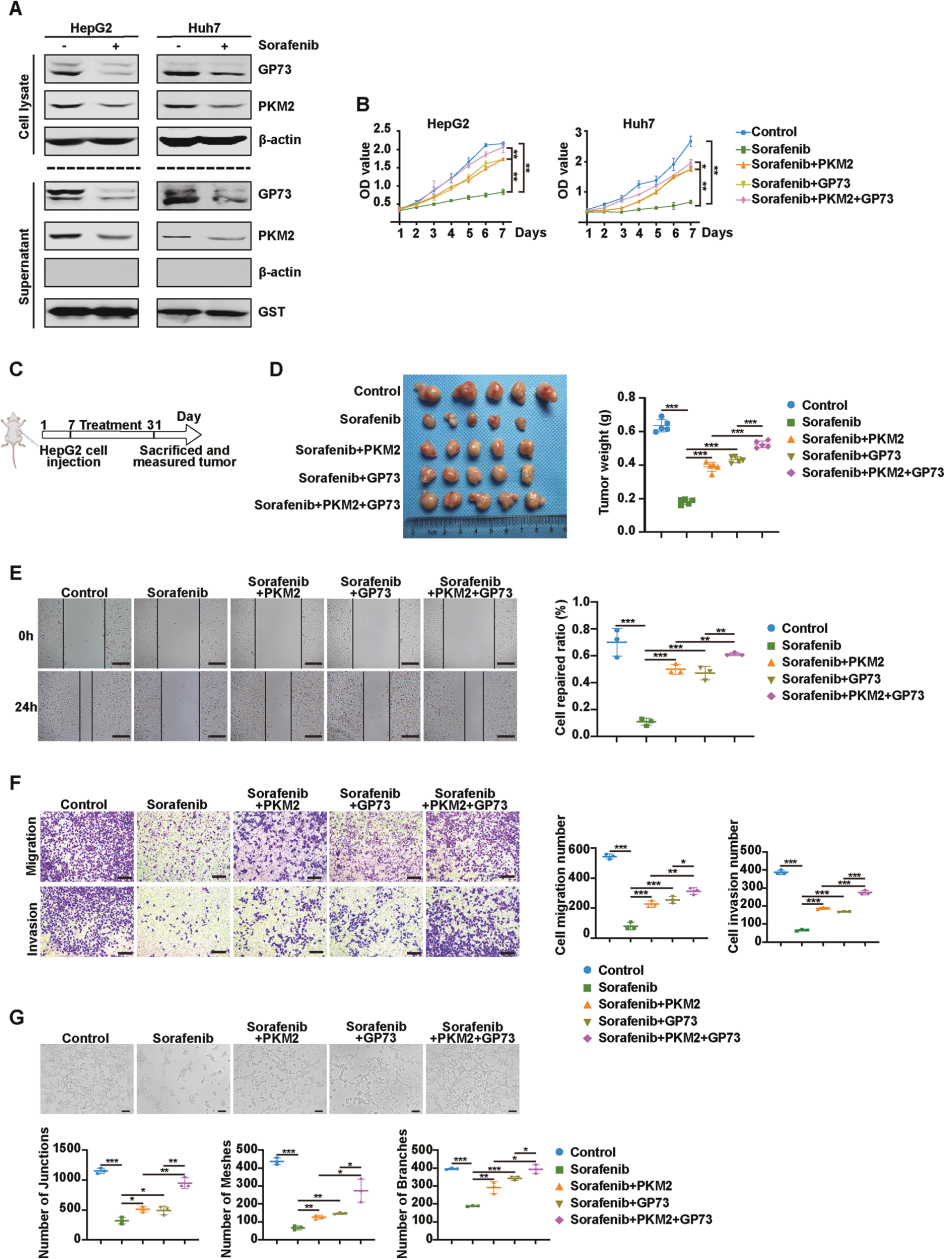
Extracellular GP73 and PKM2 attenuate sorafenib anti-tumor efficiency. **A** Sorafenib decreases the protein levels of intracellular and secreted GP73 and PKM2. The protein levels of GP73 and PKM2 in lysate and supernatant of HCC cells treated with or without sorafenib (10 μmol/L, 24 h) were measured using western blotting. β-actin was used as an intracellular loading control and GST as an extracellular loading control. **B** MTT assay showed that extracellular GP73 and PKM2 attenuated the inhibitory effect of sorafenib on the proliferation of HCC cells. **C**, **D** Extracellular PKM2 and GP73 synergistically resist the anti-tumor effects of sorafenib on HCC cells. **C** The schematic description of the in vivo experiment. HepG2 cells were subcutaneously injected into BALB/c nude mice. When the tumors were established, the mice were treated with sorafenib (30 mg/kg/day) orally. PKM2 or GP73 was given via intraperitoneal injection at a dose of 10 mg/kg twice a week. **D** Left, the image of tumors from the indicated groups. Right, the tumors were weighed after HepG2 cells injection for 31 days. Data were represented as the mean ± SD of five mice for each group. **E** Left, representative images of the wound healing assay reveal the migration potential of HUVEC after incubation with the indicated treatment. Scale bar, 500 μm. Right, the quantitative statistic of cell repaired ratio measured by Image J. *n* = 3 per each group. **F** Left, representative images of the Transwell assay show the migration and invasion potential of HUVEC after incubation with the indicated treatment. Scale bar, 200 μm. Right, the statistical graph of cell migration and invasion number. *n* = 3 per each group. **G** Top, representative images of the tube formation assay reveal the tube-forming ability of HUVEC after incubation with the indicated treatment. Scale bar, 100 μm. *n* = 3 per each group. Bottom, the quantitative statistic of tube formation measured by Image J. Error bars represent mean ± SD. *p <0.05, ** *p* <0.01, *** *p* <0.001.

Sorafenib is the first first-line drug approved by the FDA for the treatment of advanced HCC; however, many HCC patients are resistant to sorafenib [22]. Sorafenib treatment decreased the protein levels of intracellular GP73 and PKM2 as well as secreted GP73 and PKM2 (Fig. 6A).

Next, we explored whether extracellular GP73 or PKM2 affects the anti-tumor effects of sorafenib. Figure 6B indicated that HepG2 and Huh7 cells were treated with DMSO, sorafenib, PKM2+sorafenib, GP73+sorafenib, or PKM2 + GP73+sorafenib individually. The MTT assay was performed on HepG2 and Huh7 cells subjected to the treatment described above. The results showed that sorafenib significantly inhibited the growth of HepG2 and Huh7 cells, whereas secretory GP73 or PKM2 attenuated the inhibitory effect of sorafenib on the proliferation of HCC cells, and the combination of PKM2 and GP73 significantly reduced the inhibition of sorafenib. We further confirmed this result using a nude mice xenograft model of HCC cells as shown in Fig. 6C. Sorafenib inhibited tumorigenesis, whereas treatment with PKM2 or GP73 promoted the tumor growth, and the combination treatment synergized to significantly promote tumorigenesis (Fig. 6D). These data indicated that when combined extracellular GP73 and PKM2, the resistance of HCC cells to sorafenib was enhanced. Moreover, we explored whether extracellular PKM2 or GP73 weakened the inhibition of angiogenesis by sorafenib. Wound healing, Transwell, and tube formation assays of HUVEC subjected to the treatments described above were conducted (Fig. 6E–G). The results showed that sorafenib inhibited migration, invasion, and tube formation in HUVEC. Secretory PKM2 and GP73 attenuated sorafenib inhibition, and co-treatment with secretory PKM2 and GP73 synergistically attenuated the inhibitory effect of sorafenib on HUVEC migration, invasion, and tube formation. These results indicate that extracellular PKM2 or GP73 alone attenuates the inhibition of sorafenib on HCC cell proliferation and HUVEC angiogenesis. In the collective treatment with extracellular PKM2 and GP73, HCC cells and HUVEC are more resistant to sorafenib.

### Sorafenib combined with shikonin can more strongly inhibit angiogenesis and macrophage polarization by targeting GP73 and PKM2

**Fig. 7:**
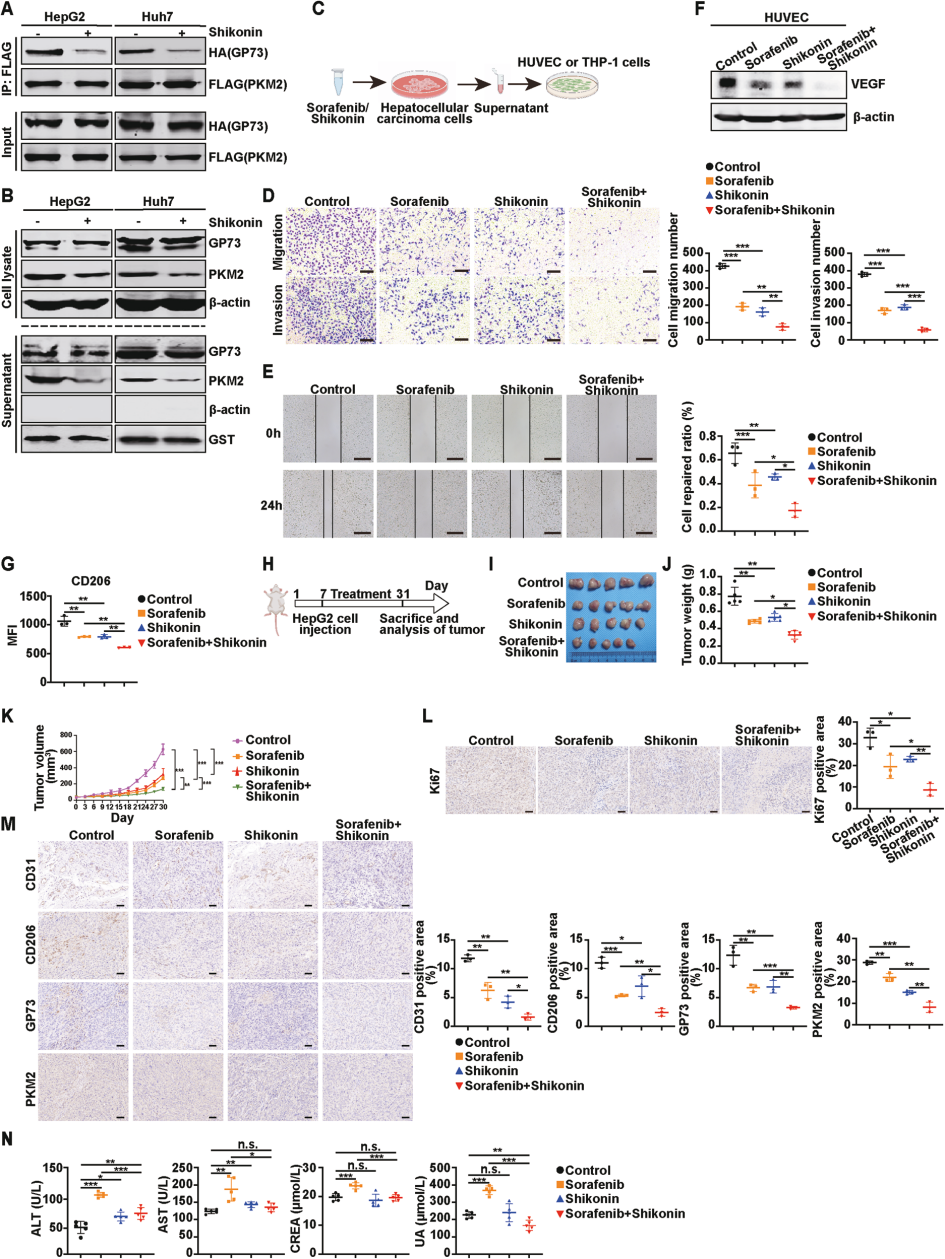
Sorafenib combined with shikonin can more strongly inhibit angiogenesis and macrophage polarization by targeting GP73 and PKM2. **A** Shikonin attenuates the intracellular interaction of GP73 and PKM2 in HCC cells. HCC cells co-transfected with HA-GP73 and FLAG-PKM2 and treated with or without shikonin (10 μmol/L, 24 h) were collected to conduct the Co-IP assay. **B** Protein levels of GP73 and PKM2 in cell lysates and supernatants of HCC cells after incubation with or without shikonin were measured by western blotting. β-actin was used as an intracellular loading control and GST as an extracellular loading control**. C** Schematic illustration for the drug combination therapy. **D**, **E** The combination therapy of sorafenib and shikonin shows a more significant inhibition on HUVEC migration and invasion. **D** Left, representative images of the Transwell assay showed the migration and invasion potential of HUVEC after incubation with the indicated supernatant from HepG2 cells. Scale bar, 200 μm. Right, the statistical graph of cell migration and invasion number. *n* = 3 per each group. **E** Left, representative images of the wound healing assay revealed the migration potential of HUVEC after incubation with the indicated supernatant from HepG2 cells. Scale bar, 500 μm. Right, the quantitative statistic of cell repaired ratio was measured by Image J. *n* = 3 per each group. **F** The protein levels of VEGF in HUVEC after incubation with the indicated supernatant from HepG2 cells were analyzed using western blotting. **G** Flow cytometry showed that the combination therapy of sorafenib and shikonin had a more significant inhibition on the MFI of the M2 macrophage gene CD206. *n* = 3 per each group. **H** Schematic description of the establishment of the animal model. **I**–**L** The combined treatment of sorafenib with shikonin suppresses tumor growth in vivo. **I** Sorafenib combined with shikonin had more significant inhibition on tumorigenesis of HepG2 cells in nude mice (five mice per group). **J** The tumors were weighed after injection for 31 days (five mice per group). **K** The tumor growth curve was shown. **L** Left, representative IHC images of the Ki67 expression in subcutaneous tumors in mice. Scale bar, 50 μm. Right, the statistical graph of the positive area of Ki67 per field. *n* = 3 per each group. **M** Left, representative IHC images of the CD31, CD206, GP73, and PKM2 expressions in subcutaneous tumors in mice. Scale bar, 50 μm. Right, the statistical graph of the positive area of CD31, CD206, GP73, and PKM2 per field. *n* = 3 per each group. **N** Content of ALT, AST, CREA, and UA from the blood of mice in the indicated groups (five mice per group). Error bars represent mean ± SD. *p <0.05, ** *p* <0.01, *** *p* <0.001; n.s., no significance.

Our study found that PKM2 or GP73 attenuates the anti-tumor effect of sorafenib, suggesting that searching for specific PKM2 or GP73 inhibitors and combining them with sorafenib may be an effective treatment for HCC. Shikonin is a potent PKM2 inhibitor derived from the Chinese medicine Arnebiae Radix with anti-inflammatory and anti-tumor activities [23]. It has been reported that shikonin inhibits the dimerization and tetramerization of PKM2 in mouse macrophages, thereby inhibiting the enzyme activity and transcriptional activation of PKM2 [24]. However, whether it inhibits the secretion of PKM2 in HCC cells remains unclear. We treated HepG2 and Huh7 cells with shikonin, and the Co-IP experiment showed that the binding of GP73 and PKM2 was significantly weakened compared with that in the control group (Fig. 7A). Cell lysates and supernatants were collected and analyzed by western blotting. Compared with the control group, both intracellular and extracellular GP73 protein levels remained unchanged, whereas intracellular and extracellular PKM2 protein levels decreased significantly (Fig. 7B). This suggests that shikonin inhibits GP73-mediated PKM2 secretion by inhibiting the binding between GP73 and PKM2. Subsequently, sorafenib and shikonin were combined to treat HCC cells, and the supernatant was collected and incubated with HUVEC and THP-1 cells for 24 h to detect the effects of the combination on angiogenesis and macrophage polarization. This process is illustrated in Fig. 7C.

Transwell (Fig. 7D) and wound healing (Fig. 7E) experiments showed that combined treatment with sorafenib and shikonin inhibited HUVEC migration more significantly than sorafenib or shikonin alone. Western blotting also showed that after combined treatment with sorafenib and shikonin, the level of VEGF protein declined most significantly (Fig. 7F). These results suggest that the combination of sorafenib and shikonin significantly inhibit angiogenesis in HUVEC. To evaluate the effect of combination therapy on macrophage polarization, the MFI of the M2 macrophage marker CD206 in THP-1 cells after treatment with the described groups was analyzed by flow cytometry (Fig. 7G, Supplementary Fig. 5). Compared with treatment with sorafenib or shikonin individually, the MFI of CD206 in THP-1 cells decreased more significantly after treatment with sorafenib and shikonin. Given the obvious therapeutic effect of sorafenib combined with shikonin, we used a mouse model to investigate whether shikonin can improve the anti-tumor effect of sorafenib on HCC in vivo, as shown in Fig. 7H. HepG2 cells were subaxillary into BALB/c nude mice, and olive oil, sorafenib, shikonin, or sorafenib+shikonin was administered orally every two days for two weeks. As shown in Fig. 7I–K, sorafenib combined with shikonin had the most significant inhibitory effect on tumor proliferation compared with the control, sorafenib, or shikonin treatment groups.

Moreover, the expression of Ki67 in the sorafenib combined with shikonin group was significantly lower than that in the sorafenib alone group (Fig. 7L). Next, IHC staining of the tumors was performed to evaluate the effect of the treatment on angiogenesis and macrophage polarization, and to investigate whether the treatment could target GP73 and PKM2. Importantly, the combination treatment group with sorafenib and shikonin had the smallest and weakest CD31 positive staining area, indicating that this treatment significantly inhibited HCC angiogenesis. The IHC staining results for CD206, GP73, and PKM2 were the same (Fig. 7M). These results suggest that the combination of sorafenib and shikonin can more effectively inhibit angiogenesis and macrophage polarization, and this treatment targets GP73 and PKM2. Moreover, blood biochemical analysis of liver and kidney function indicated that treatment with sorafenib alone led to hepatotoxicity and renal toxicity. However, combined treatment with sorafenib and shikonin significantly reduced hepatotoxicity and renal toxicity (Fig. 7N).

As noted above, our results indicate that sorafenib combined with shikonin strongly inhibits proliferation, angiogenesis, and M2-type macrophage polarization in HCC. Furthermore, the addition of shikonin significantly reduced hepatotoxicity and renal toxicity compared to sorafenib treatment of HCC. Our findings provide a new therapeutic strategy for the treatment of HCC using sorafenib combined with shikonin.

## Discussion

HCC, a highly lethal disease, is the most commonly diagnosed liver cancer. The poor survival rate is mainly related to late diagnosis of HCC and high drug resistance. The development of more sensitive and specific serum biomarkers for early detection and drug targeting may improve patient survival. Here, we demonstrate that secreted GP73 and PKM2 are promising biomarkers for the diagnosis and treatment of HCC. GP73 interacts with PKM2 to promote SUMO1 modification of PKM2 via enhancing the interaction of Ubc9 and PKM2, and this SUMO1 modification of PKM2 promotes the binding of GP73 and PKM2 in turn, thereby continuously facilitating the transport of PKM2 from cytoplasm to PM and eventual secretion. Secreted GP73 and PKM2 synergistically promote angiogenesis and M2-type macrophage polarization by regulating the expression of related proteins, thereby promoting the HCC progression. Moreover, macrophage polarization promotes liver fibrosis and ECM formation in HCC. Extracellular GP73 and PKM2 enhance sorafenib resistance in HCC cells. Chinese medicine-shikonin can specifically inhibit GP73-mediated PKM2 secretion and increase the sensitivity of HCC cells to sorafenib. The combination of these two drugs exerts a stronger anti-tumor effect. Collectively, our study illustrated that GP73-mediated secretion of PKM2 and GP73 promotes HCC progression by remodeling the TME, and GP73 and PKM2 can act as new therapeutic targets for HCC.

Previous studies have found that PKM2 is highly expressed in the serum of patients with HCC, which is correlated with disease progression, and serum PKM2 is expected to become a diagnostic marker for HCC [17]. However, the mechanisms underlying PKM2 secretion remain unclear. In this study, we demonstrated that high GP73 expression promotes PKM2 secretion, and GP73 secretion is independent of PKM2. Next, we studied the mechanism by which GP73 promotes secretion. Previous studies have reported that GP73 can promote the secretion of target proteins, such as AFP, MMP7, and MMP2 [7–9]. Likewise, we demonstrated that GP73 interacts with PKM2 both in vivo and in vitro, and that PKM2 secretion depends on its binding to GP73. So why does the combination of GP73 and PKM2 facilitate the secretion of PKM2? It has been reported that SUMO1 modification of PKM2 promotes the movement of PKM2 from the cytoplasm to the PM, and finally secretion [13]. Our data are consistent with these results. GP73 promotes SUMO1 modification of PKM2 in HCC cells, leading to PKM2 extracellular secretion. SUMOylation is a biochemical process catalyzed by the E1 activation enzyme, E2 conjugation enzyme, and E3 ligase, of which the only E2 conjugation enzyme-Ucb9 is highly expressed in HCC cells [25, 26]. The combination of GP73 and PKM2 promotes the translocation of PKM2 from the cytoplasm to the PM by enhancing Ubc9-mediated SUMO1 modification of PKM2. Furthermore, the interaction of GP73 and PKM2 and SUMO1 modification of PKM2 form a loop that leads to the continuous secretion of PKM2 and GP73.

In many cases, HCC is caused by chronic liver inflammation, which leads to the formation of a complex TME composed of immune and stromal cells. The TME of patients with HCC is a challenge for diagnosis and treatment, as it involves metastasis and the development of drug resistance. Therefore, exploring the complex factors that influence the formation of TME will provide new possibilities for designing novel and more effective combinatorial therapies to overcome drug resistance. Our study is the first to demonstrate that extracellular GP73 and PKM2 synergistically promote angiogenesis and macrophage polarization. Extracellular GP73 and PKM2 synergistically reduce HUVEC adhesion and promote migration, invasion, and tube formation. In addition, extracellular GP73 and PKM2 synergistically promote the differentiation of monocytes into M2 macrophages. Moreover, crosstalk between M2 macrophages and LX2 or HCC cells promotes hepatic fibrosis and remodels the ECM. This finding is consistent with the discovery by Cao et al. that GP73 may be a target for anti-liver fibrosis [27]. Hepatic fibrosis is an abnormal wound-healing response characterized by the cumulative synthesis and deposition of ECM [28]. Collagen, fibronectin, elastin, laminin, and hyaluronic acid are the main ECM proteins [29]. HSCs are the primary cells that produce ECM proteins, and their activation plays an important role in the development of liver fibrosis [30]. HSC activation leads to the expression of α-SMA [31]. Consistently, our results showed that GP73 overexpression led to significant polarization of M2 macrophages, resulting in increased expression of α-SMA in LX2 cells, whereas GP73 overexpression and simultaneous PKM2 knockdown led to decreased polarization of M2 macrophages, resulting in decreased expression of α-SMA in LX2 cells. ECM proteins showed the same results. Notably, our study revealed novel functions for extracellular GP73 and PKM2 in HCC and that extracellular GP73 and PKM2 synergistically promote HCC progression. Our findings also highlight that targeting GP73 and PKM2 may inhibit crosstalk between HCC cells and other stromal cells, thus becoming a new therapeutic strategy for HCC.

Sorafenib is the first-line drug for the treatment of HCC, but it is still associated with high mortality and poor prognosis due to drug resistance [32]. Our results showed that extracellular GP73 and PKM2 synergistically attenuated the anti-tumor efficiency of sorafenib, suggesting that high secretion of GP73 and PKM2 can lead to resistance to sorafenib. Thus, we speculate that pharmacological targeting of GP73 and PKM2 may increase the sensitivity of HCC cells to sorafenib and improve patient prognosis and survival. To test this hypothesis, we used shikonin, a potent PKM2 inhibitor derived from the traditional Chinese medicine Arnebiae Radix with anti-inflammatory and anticarcinogenic activities, in combination with sorafenib to treat HCC. Shikonin reduced the extracellular secretion of PKM2 by inhibiting the binding of GP73 and PKM2, thus further enhancing the inhibitory effect of sorafenib on angiogenesis and M2 macrophage polarization and improving the anti-tumor effect of sorafenib. Compared with other emerging combination therapies, our study has made significant progress. Nearly half of the clinical trials studying HCC combination therapy involve immune checkpoint blockades plus multikinase inhibitors [33]. However, this combination therapy comes at the cost of increased toxicities and tends to have higher adverse effects rates [34]. Our study has made significant progress, showing that shikonin reduced the hepatorenal toxicity of sorafenib. Besides, only approximately 70% of HCC patients express a high level of AFP [35], so targeting AFP in treatment HCC is not effective. GP73 played a key role in the early stages of liver disease, such as hepatitis, non-alcoholic fatty liver disease, as well as PKM2 [6, 36]. Thus, targeting GP73 and PKM2 has more therapeutic potential. Overall, our work supports a regimen in which the treatment of HCC with sorafenib and shikonin targeting GP73 and PKM2 is highly effective. In this study, we only used the HepG2 cell line as our subcutaneous mouse model to evaluate the effect of combination therapy was not enough, because sorafenib and shikonin-induced hepatotoxicity may not be accurately reflected in the HCC model. HepG2 cell line shows only partially similar genome, transcriptome, and proteome profiles as the HCC model [37]. It has a low basal activity of cytochrome P450 (CYP), which can be activated by some drugs and induce liver injury [38]. In future studies, it is necessary to use multiple cell lines as the research model to capture the HCC heterogeneity and complexity.

In summary, our study suggests that serum GP73 and PKM2 are specific biomarkers for HCC diagnosis and therapy and that the secretion of GP73 and PKM2 promotes HCC progression and drug resistance by promoting angiogenesis and M2-like macrophage polarization. The combination of sorafenib and shikonin targeting GP73 and PKM2 could be used as an effective treatment for HCC. Our study provides new drug targets and treatment options for HCC.

## Supplementary information

Supplementary Figures

Supplementary Tables

Original western blots

## Data Availability

All data and materials are available in the main text and supplementary information.
