# Correction: GP73-mediated secretion of PKM2 and GP73 promotes angiogenesis and M2-like macrophage polarization in hepatocellular carcinoma

**DOI:** 10.1038/s41419-025-07628-7

**Published:** 2025-05-16

**Authors:** Shujie Wang, Tongjia Zhang, Yue Zhou, Zitao Jiao, Kejia Lu, Xinyi Liu, Wei Jiang, Zhe Yang, Hui Li, Xiaowei Zhang

**Affiliations:** 1 Department of Biochemistry and Molecular Biology, School of Basic Medical Sciences, Beijing Key Laboratory of Protein Posttranslational Modifications and Cell Function, Peking University Health Science Center, Beijing, 100191 China; 2 Department of Pathology, The First Affiliated Hospital of Kunming Medical University, Kunming, 650032 Yunnan China

**Keywords:** Diagnostic markers, Cancer microenvironment, Tumour biomarkers

Correction to: *Cell Death and Disease* 10.1038/s41419-025-07391-9, published online 5 February 2025

We regret that an error was made in the original version of Figure 6F. During the assembly of the figure, the migration image for the sorafenib+GP73 condition was inadvertently misused. This mistake has been corrected, and a revised version of Figure 6F, with the correct image, is provided below.

**Amended Figure 6F** 
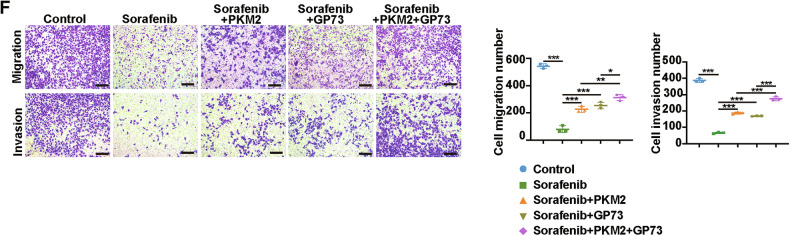


**Original Figure 6F** 
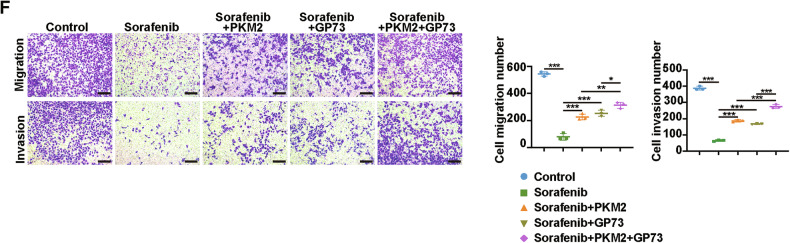


We would like to emphasize that the scientific conclusions of our study remain unchanged and unaffected by this unintentional error. All authors agree with this revision request. We sincerely apologize for any confusion this may have caused.

The original article has been updated.

