# Supplementary material for: GP73-mediated secretion of PKM2 and GP73 promotes angiogenesis and M2-like macrophage polarization in hepatocellular carcinoma
Source: Cell Death Dis. 2025 Feb 5;16(1):69. doi: 10.1038/s41419-025-07391-9 (PMC11794714; doi:10.1038/s41419-025-07391-9)
Supplement: Supplementary file 1 — Supplementary Figures [file 41419_2025_7391_MOESM1_ESM.pdf]

Supplementary Material

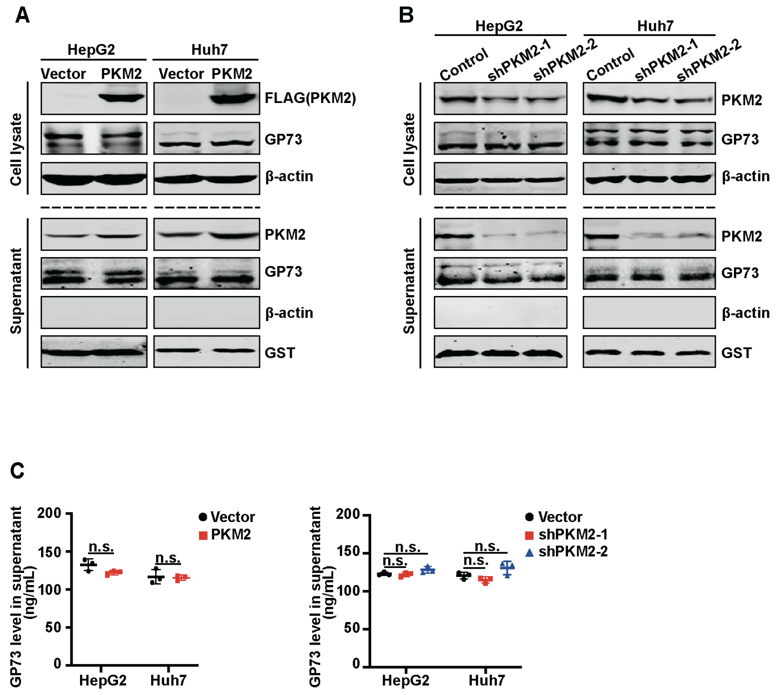

Supplementary Figure 1. PKM2 does not promote GP73 secretion. The protein levels of GP73 in cell lysates and supernatants after PKM2 overexpression or knockdown in HCC cells were measured by western blotting (**A**, **B**) and ELISA (**C**). β-actin was used as an intracellular loading control, and GST as an extracellular loading control. n=3 per each group. n.s., no significance**.**

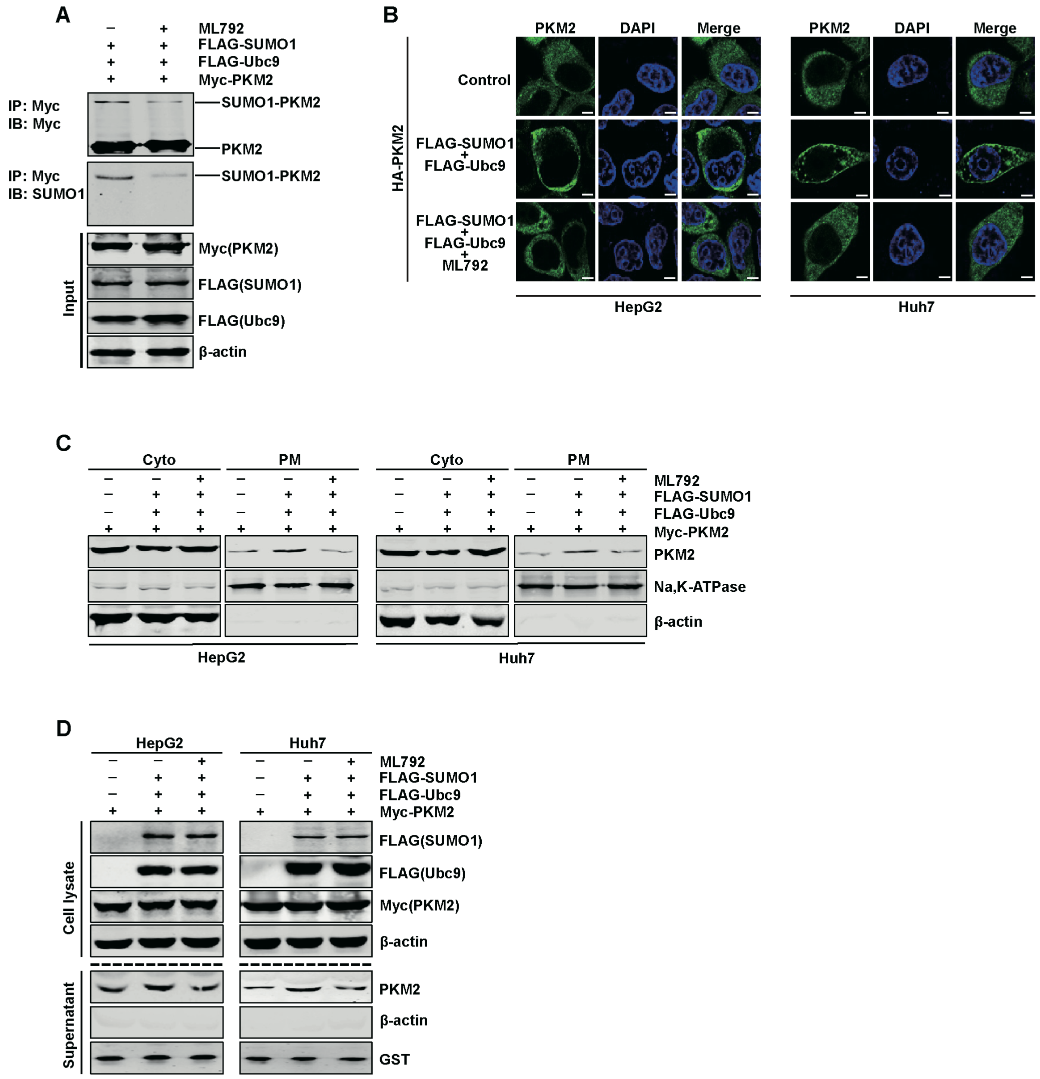

Supplementary Figure 2. The inhibition of SUMO1 modification attenuates PKM2 secretion. **A** SUMO1 modification inhibitor ML792 attenuates the SUMO1 modification of PKM2 in Huh7 cells. Huh7 cells co-transfected with FLAG-SUMO1, FALG-Ubc9, Myc-PKM2 and treated with or without ML792 were subjected to an *in vivo* SUMO1 conjugation assay. B, C ML792 decreases the PM location of PKM2 in HCC cells. IF (**B**) and western blotting (**C**) were conducted with HCC cells after transfected with the indicated plasmids and treated with or without ML792. Scale bar, 5 μm. Na, K-ATPase was used as a PM protein loading control and β-actin was used as a cytoplasm loading control. **D** ML792 attenuates PKM2 secretion. The protein levels of PKM2 in the cell lysate and supernatant after the indicated treatment in HCC cells were determined by western blotting. β-actin was used as an intracellular loading control, and GST was used as an extracellular loading control.

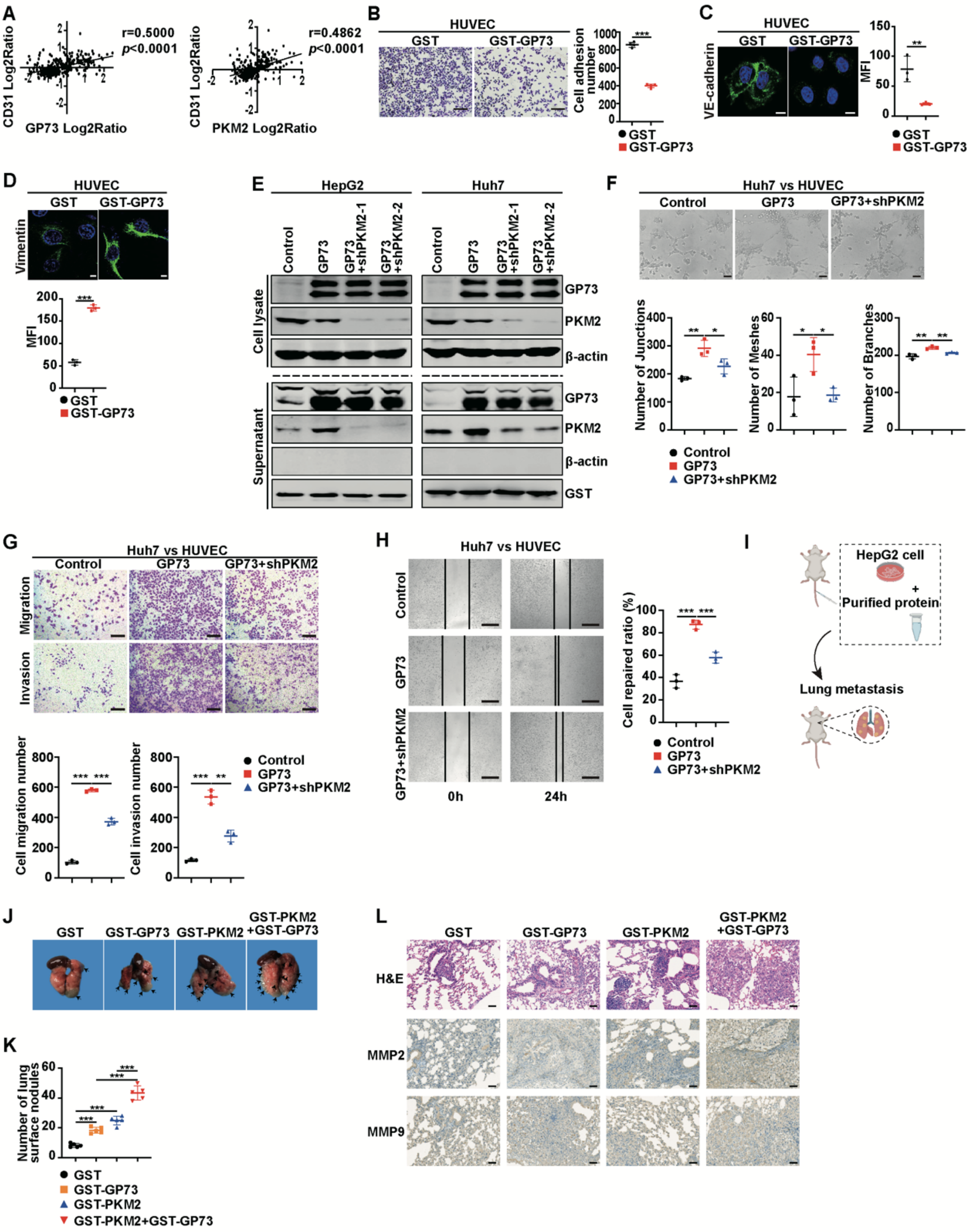

Supplementary Figure 3. **A** Pearson's correlation analysis of GP73 and CD31, PKM2 and CD31 expression in HCC tissues from the CPTAC database. **B** Representative cell adherence assay images showed that GST-GP73 decreased the number of adherent HUVEC. Scale bar, 200 μm. n=3 per each group. **C** Left, representative IF merged images revealed that GST-GP73 decreased the VE-cadherin expression in HUVEC. VE-cadherin (green), DAPI (blue), nucleus. Scale bar, 10μm. Right, the statistical evaluation of the MFI. n=3 per each group. **D** Top,

representative IF merged images revealed that GST-GP73 increased the Vimentin expression in HUVEC. Vimentin (green), DAPI (blue), nucleus. Scale bar, 5 μm. Bottom, the statistical evaluation of the MFI. n=3 per each group. **E** PKM2 knockdown decreases GP73-mediated PKM2 secretion. The protein levels of GP73 and PKM2 in cell lysates and supernatants after HCC cells stably expressed GP73 with or without PKM2 knockdown were measured using western blotting. β-actin was used as an intracellular loading control, and GST was used as an extracellular loading control. **F** Top, representative images of the tube formation assay revealed the tube-forming ability of HUVEC treated with the indicated group supernatant of Huh7 cells. Scale bar, 100 μm. Bottom, the quantitative statistic of tube formation measured by Image J. n=3 per each group. **G** Top, representative images of the Transwell assay showed the migration and invasion potential of HUVEC treated with the indicated group supernatant of Huh7 cells. Scale bar, 200 μm. Bottom, the statistical graph of cell migration and invasion number. n=3 per each group. **H** Left, representative images of the wound healing assay revealed the migration potential of HUVEC treated with the indicated group supernatant of Huh7 cells. Scale bar, 500 μm. Right, the quantitative statistics of cell repaired ratio was measured by Image J. n=3 per each group. **I-L** Extracellular GP73 and PKM2 promote metastasis of HCC cells. **I** Schematic description of the establishment of the lung metastasis model. HepG2 cells and the indicated purified protein were injected into the tail vein of nude mice. **J** Top, representative images of metastatic lung tumors are shown. Arrows indicate metastatic lung tumors. **K** Visible lung tumors were analyzed (five mice per group). **L** Images of H&E staining, IHC of MMP2 and MMP9 of metastatic lung tumors. Error bars represent mean ± SD. *$p < 0.05$, ** $p < 0.01$, *** $p < 0.001$.

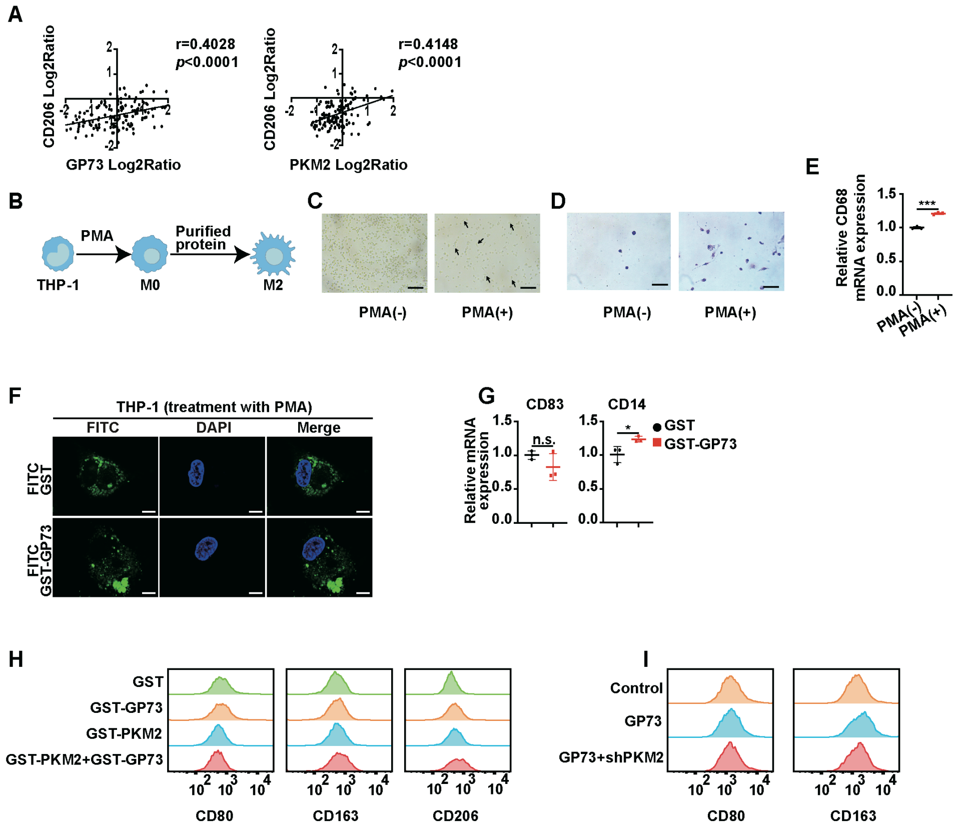

Supplementary Figure 4. A Pearson's correlation analysis of GP73 and CD206, PKM2, and CD206 expression in HCC tissues from the CPTAC database. **B** Schematic diagram of THP-1 cells treated with PMA and purified protein. THP-1 cells treated with PMA after 24 h were captured (**C**) and cell adherence assay was conducted (**D**). Scale bar, 200 μm. **E** PMA induces THP-1 cells into M0 macrophages. The relative mRNA levels of CD68 in THP-1 cells after incubation with PMA were measured by Real-time PCR. n=3 per each group. **F** Representative IF merged images showed that the FITC-labelled purified GP73 protein was phagocytized by THP-1 cells. Scale bar, 10μm. **G** GST-GP73 induces THP-1 cells into macrophages, not DC. The relative mRNA expressions of DC marker CD83 and macrophage marker CD14 were evaluated using Real-time PCR after THP-1 cells incubation with GST or GST-GP73. n=3 per each group. **H** Flow cytometry was used to measure the fluorescence intensity of THP-1 cells after the indicated protein treatment. **I** Flow cytometry was used to measure the fluorescence intensity of THP-1 cells after the indicated supernatant treatment. Error bars represent mean ± SD. *$p < 0.05$, *** $p < 0.001$; n.s., no significance.

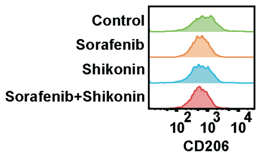

Supplementary Figure 5. Flow cytometry was used to measure the fluorescence intensity of THP-1 cells after the indicated treatment.
