# Supplementary material for: GP73-mediated secretion of PKM2 and GP73 promotes angiogenesis and M2-like macrophage polarization in hepatocellular carcinoma
Source: Cell Death Dis. 2025 Feb 5;16(1):69. doi: 10.1038/s41419-025-07391-9 (PMC11794714; doi:10.1038/s41419-025-07391-9)
Supplement: Supplementary file 2 — Supplementary Tables [file 41419_2025_7391_MOESM2_ESM.docx]

Supplementary Tables

Supplementary Table 1. Primers for Real-time PCR

|  | Forward | Reverse |
| --- | --- | --- |
| *PKM2* | ACTCGGGCTGAAGGCAGTGA | TGTGGGGTCGCTGGTAATGG |
| *CD83* | TCCTGAGCTGCGCCTACAG | GCAGGGCAAGTCCACATCTT |
| *CD14* | TTCTGAGGGTCCTCGTCAAC | CGTGTGGATCCTGAGGGTTA |
| *CD80* | TGGTGCTGGCTGGTCTTTC | CTGTGCCACTTCTTTCACTTCC |
| *iNOS* | TTCAGTATCACAACCTCAGCAAG | TGGACCTGCAAGTTAAAATCCC |
| *CD163* | AGTCTGCTCAAGATACACAGAAA | GGTAGAAAGGGCAACTCCACA |
| *CCL5* | TCGTCCACAGGTCAAGGATG | CACACACTTGGCGGTTCTTT |
| *IL-10* | CTGTTGCCTGGTCCTCCTG | TTCTCAGCTTGGGGCATCAC |
| *CD206* | ATCACGAAGCCAAGGTCCAG | GTGGGTGAACCGAACCTCTT |
| *CD68* | GCAATAAGCACCAGGGCGAGGAGGC | TACAATGTGTCCTTCCCCCACGCAG |
| *GAPDH* | CCATGGAG AAGGCTGGGG | CAAAGTTGTCATGGATGACC |

Supplementary Table 2. Antibody information

| Primary antibody | Source | Catalog number |
| --- | --- | --- |
| Anti-GP73 for western blotting | Santa Cruz | sc-365817 |
| Anti-GP73 for IF, IHC | Proteintech | 66331-1-Ig |
| Anti-PKM2 for western blotting, IF, IHC | Proteintech | 15822-1-AP |
| Anti-GST for western blotting | Proteintech | 10000-0-AP |
| Anti-Collagen I for western blotting, IHC | Proteintech | 14695-1-1AP |
| Anti-FLAG for western blotting | Sigma | F1804 |
| Anti-αSMA for western blotting, IHC | BioLegend | 614852 |
| Anti-HA for western blotting, IF | BioLegend | 16B12 |
| Anti-His for western blotting | Proteintech | 66005-1-Ig |
| Anti-Na, K-ATPase for western blotting, IF | CST | 23565 |
| Anti-SUMO1 for western blotting | Santa Cruz | sc-5308 |
| Anti-Ubc9 for western blotting | Proteintech | 10070-1-AP |
| Anti-VE-cadherin for western blotting, IF | Santa Cruz | sc-9989 |
| Anti-Vimentin for western blotting, IF | Santa Cruz | sc-6260 |
| Anti-N-cadherin for western blotting | CST | 13116 |
| Anti-E-cadherin for western blotting | CST | 14472 |
| Anti-MMP2 for western blotting | CST | 40994 |
| Anti-MMP9 for western blotting | Proteintech | 10375-2-AP |
| Anti-VEGF for western blotting | Santa Cruz | sc-7269 |
| Anti-CD80 for flow cytometry | BioLegend | 2D10 |
| Anti-CD163 for flow cytometry | BioLegend | GHI/61 |
| Anti-CD163 for IF | CST | 93498 |
| Anti-CD206 for flow cytometry | BioLegend | 15-2 |
| Anti-Ki67 for immunohistochemistry | Affinity Biosciences | AF0198 |
| Anti-β-actin | Proteintech | 66009-1-Ig |
| Secondary antibody | Source | Catalog number |
| DyLight 800 Conjugated anti-Rabbit IgG | EarthOx | E032820 |
| DyLight 800 Conjugated anti-Mouse IgG | EarthOx | E032810 |
| Goat Anti-Mouse IgG (H+L), Dylight549 | EarthOx | E032310 |
| Goat Anti-Mouse IgG (H+L), Dylight649 | EarthOx | E032620 |
| Goat Anti- Rabbit IgG (H+L), Dylight488 | EarthOx | E032210 |
