# Supplementary figures and images for: GP73-mediated secretion of PKM2 and GP73 promotes angiogenesis and M2-like macrophage polarization in hepatocellular carcinoma
Source: Cell Death Dis. 2025 Feb 5;16(1):69. doi: 10.1038/s41419-025-07391-9 (PMC11794714; doi:10.1038/s41419-025-07391-9)

Figure 1

H

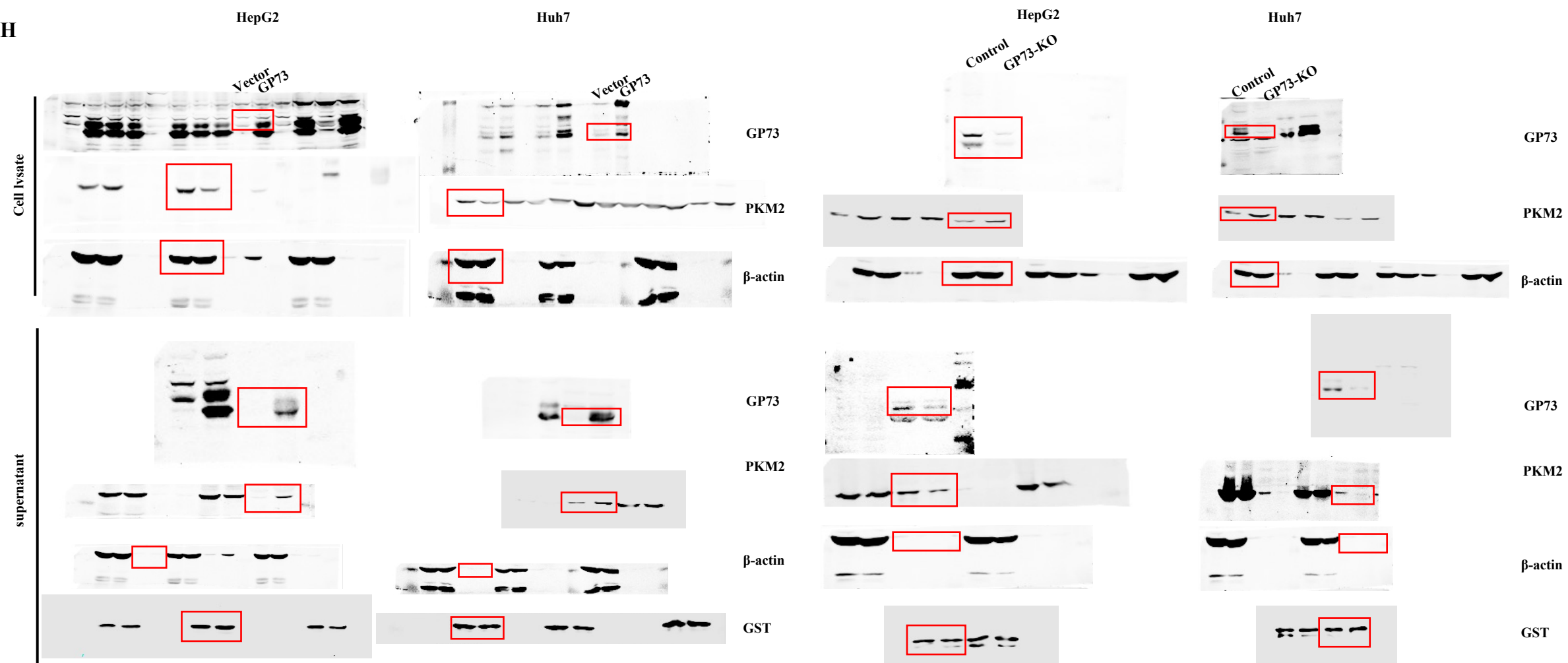

**Figure 2**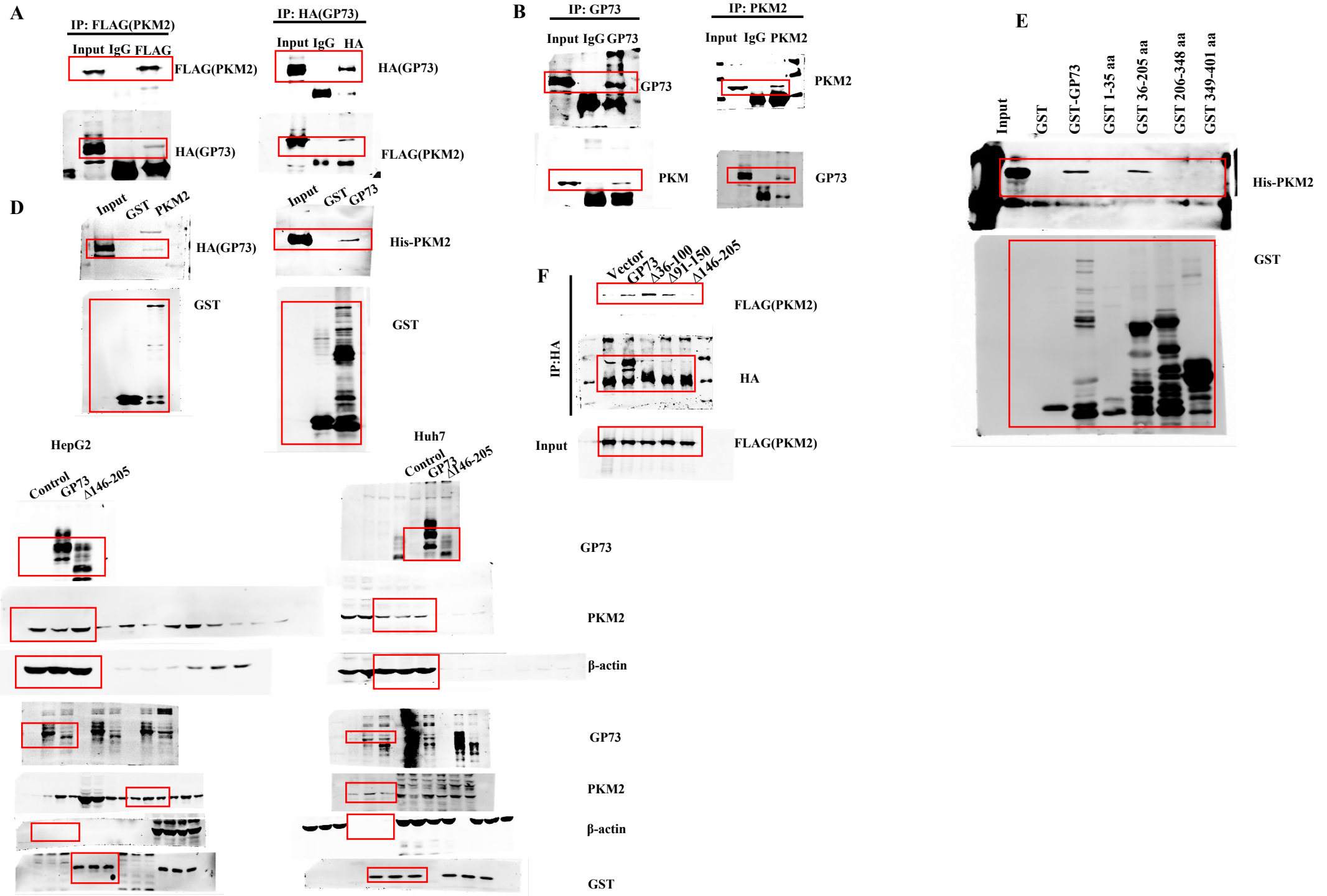



**Figure 4**

**B**

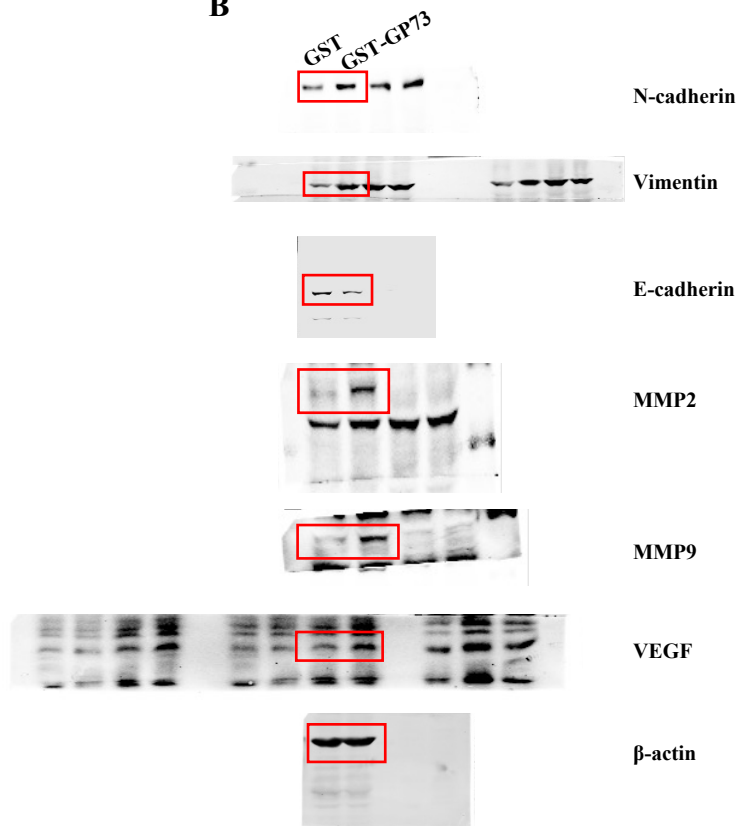

Figure 5

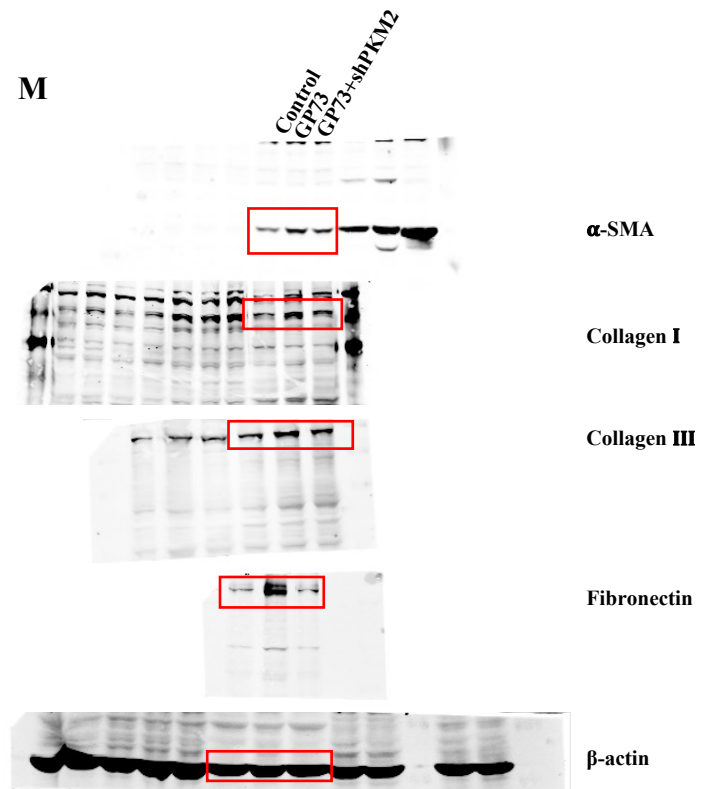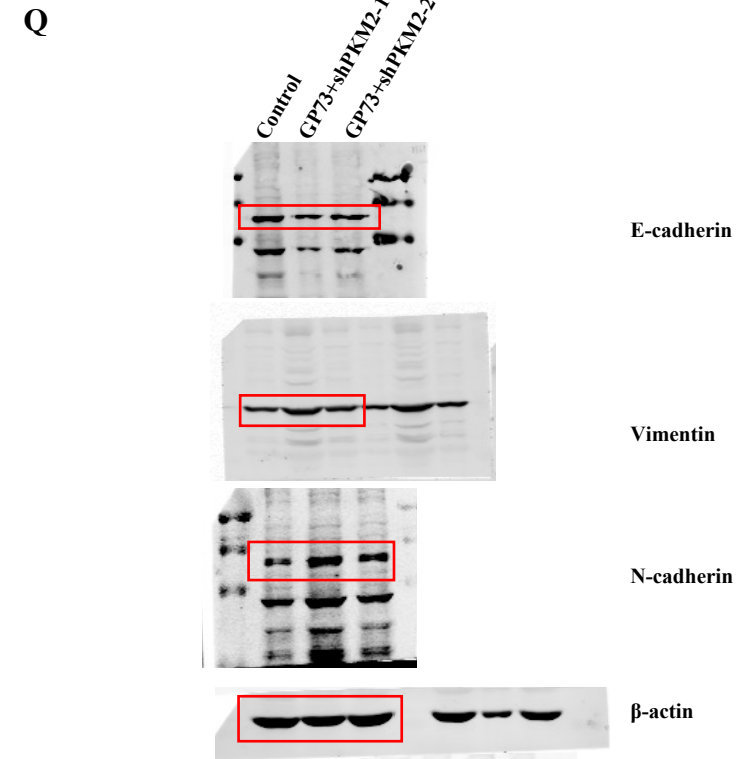

**Figure 6**

**B**

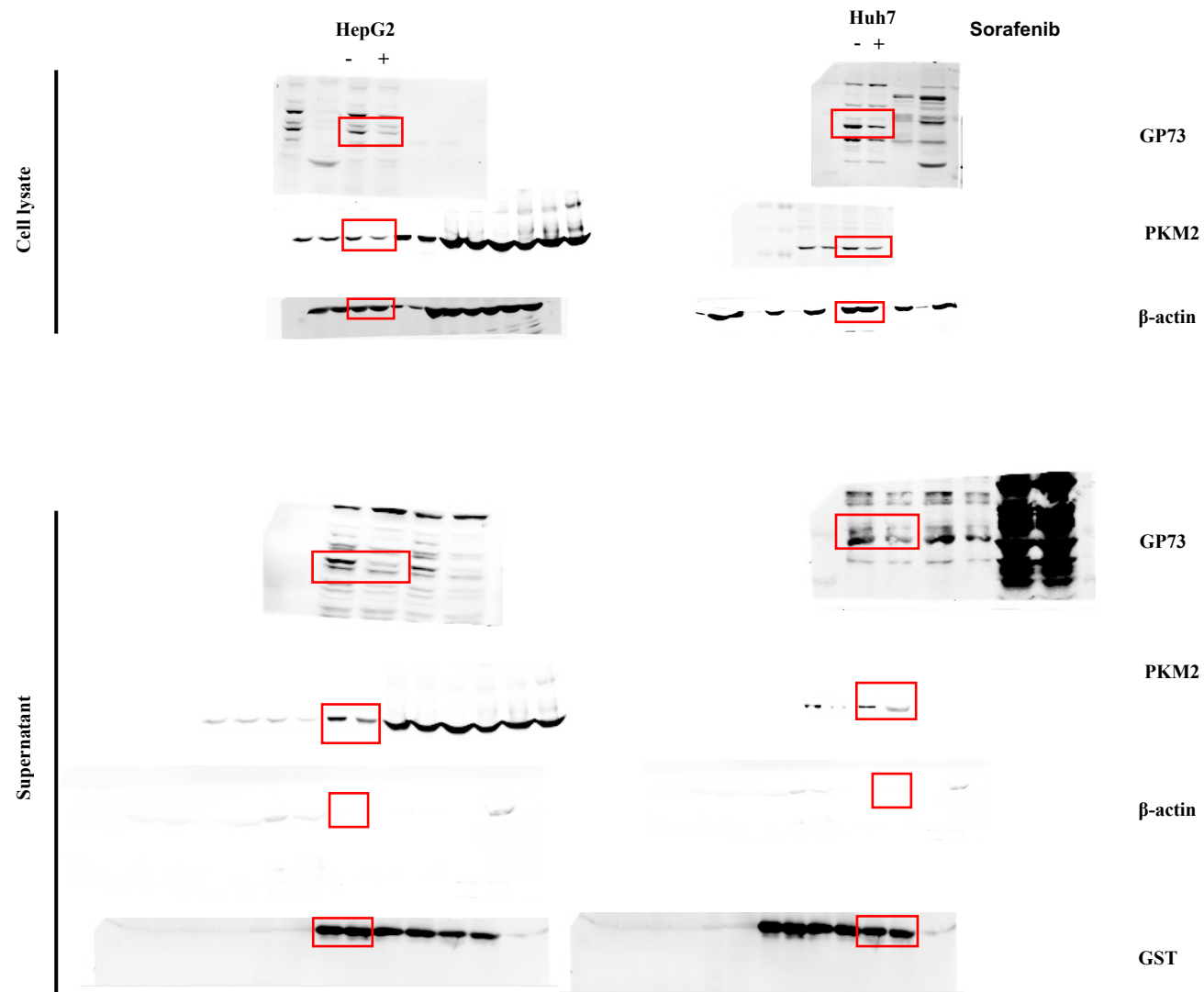

**Figure 7**

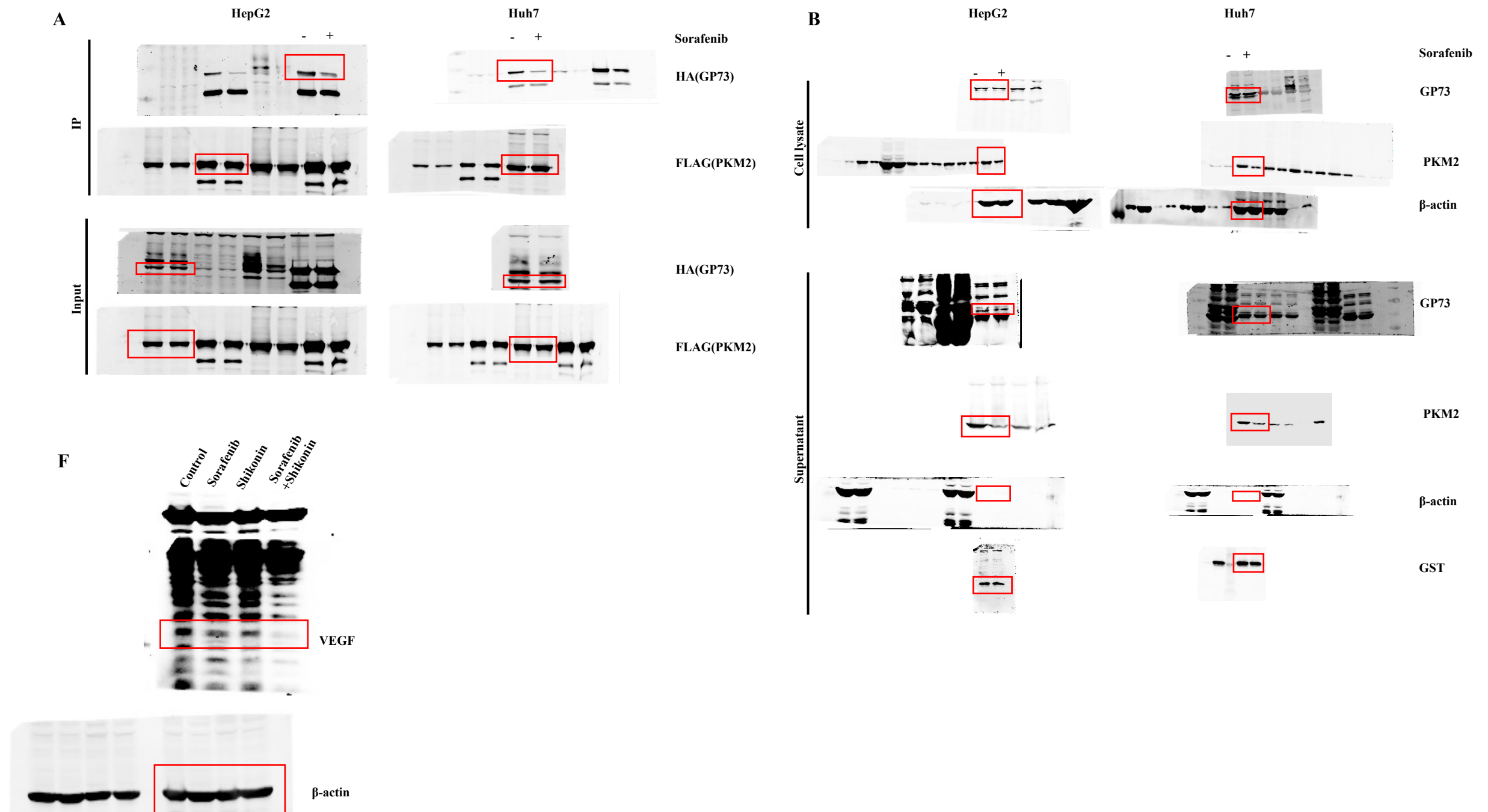

Figure S1

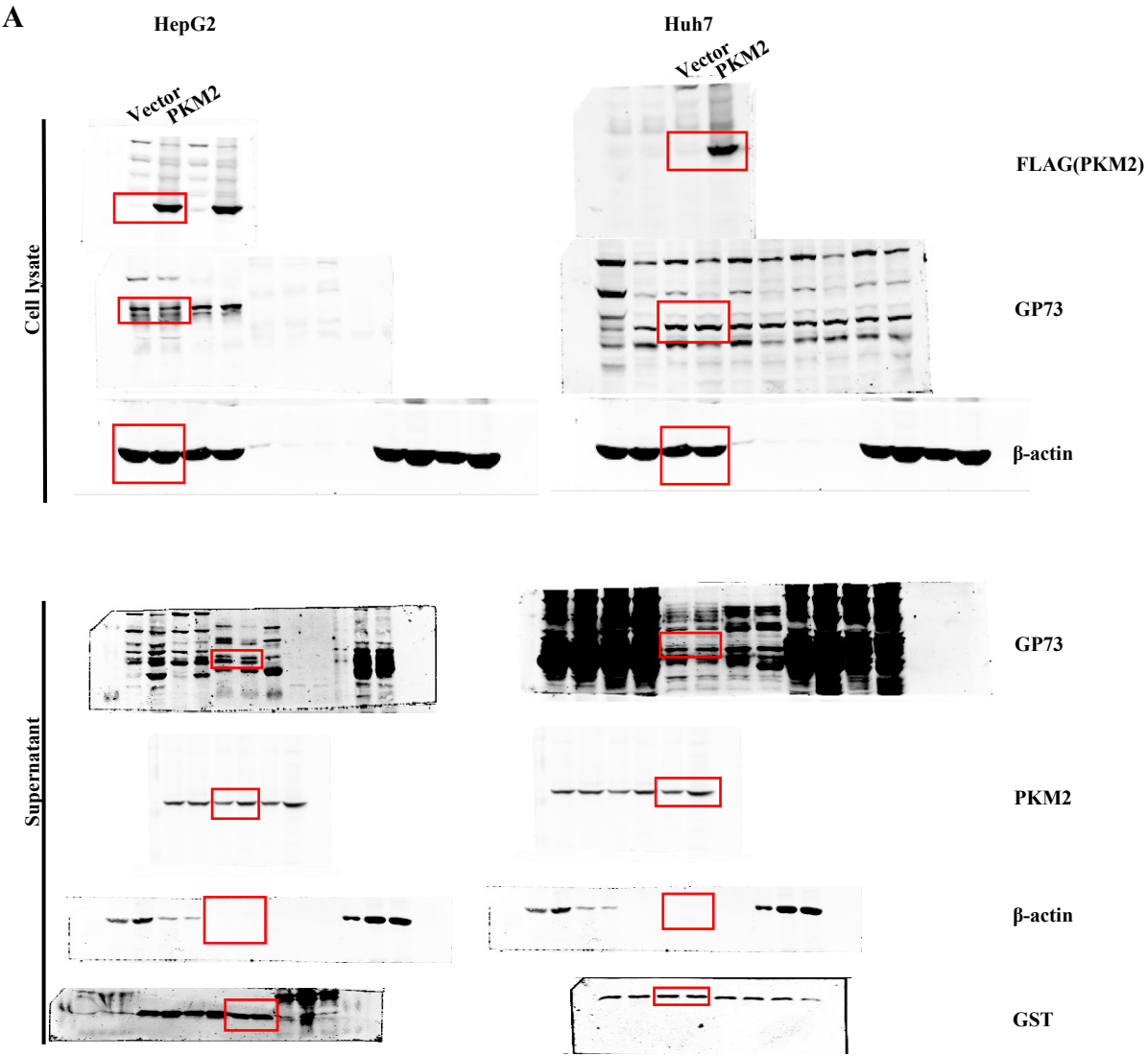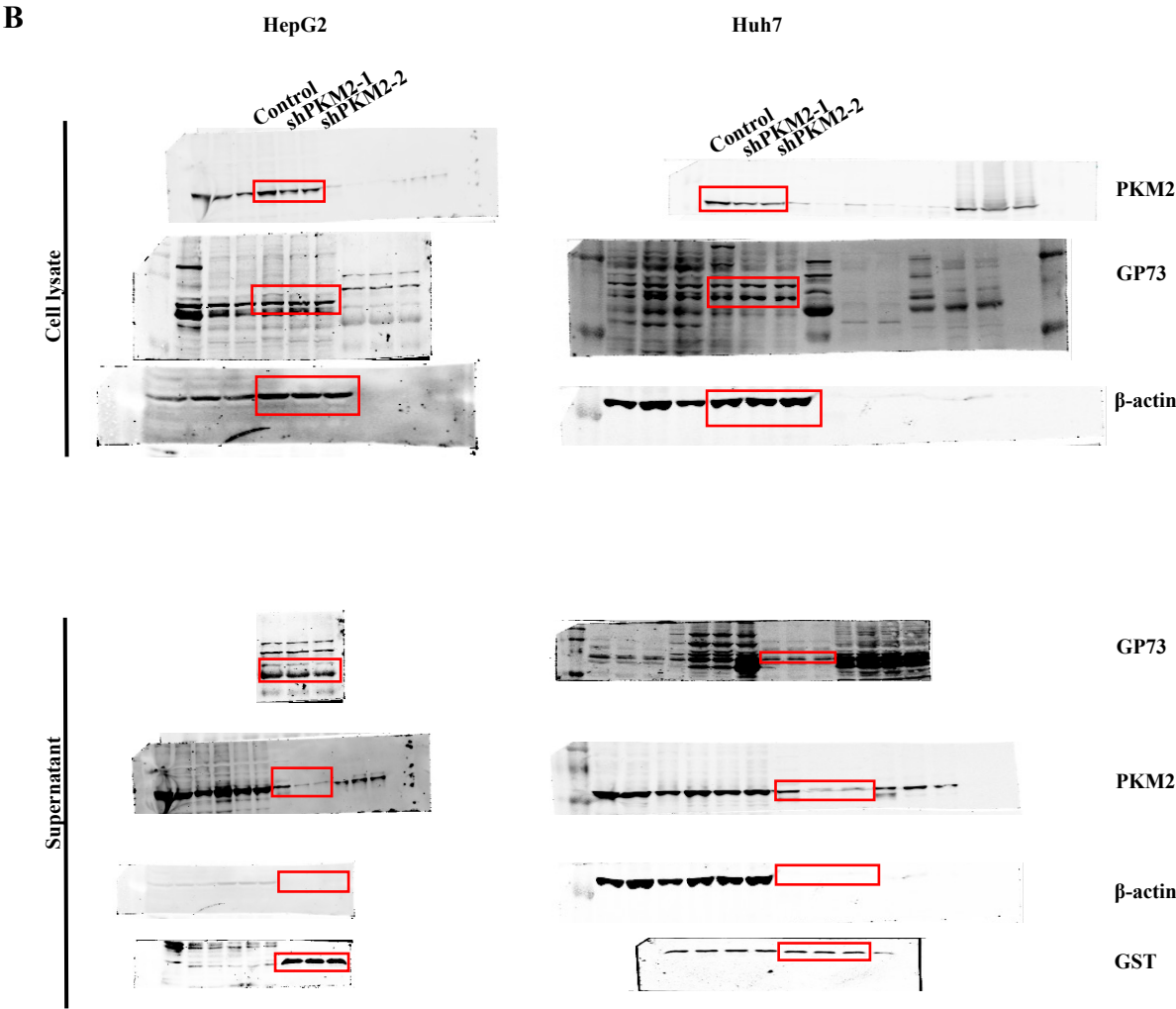

Figure S2

A

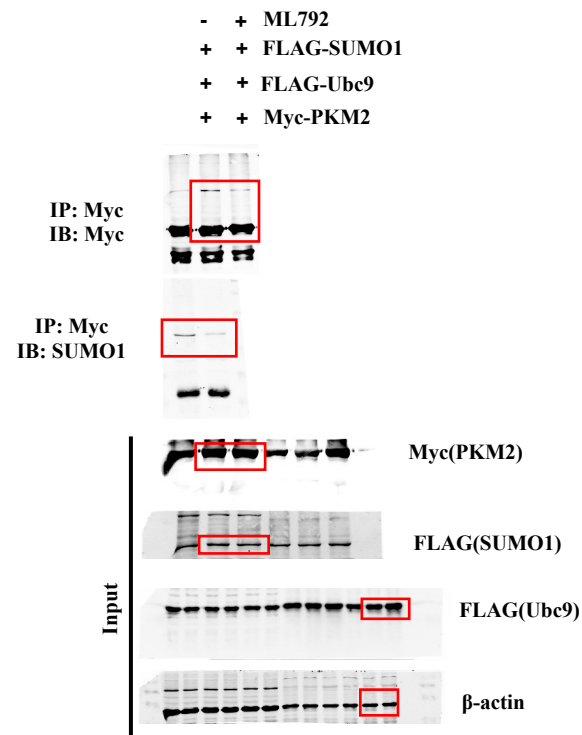

C

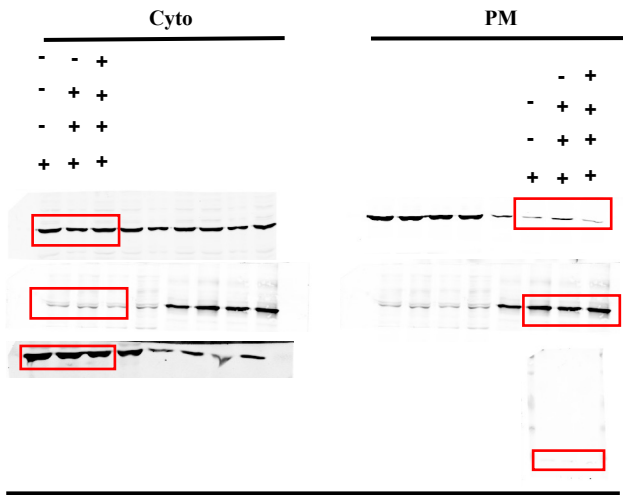

D

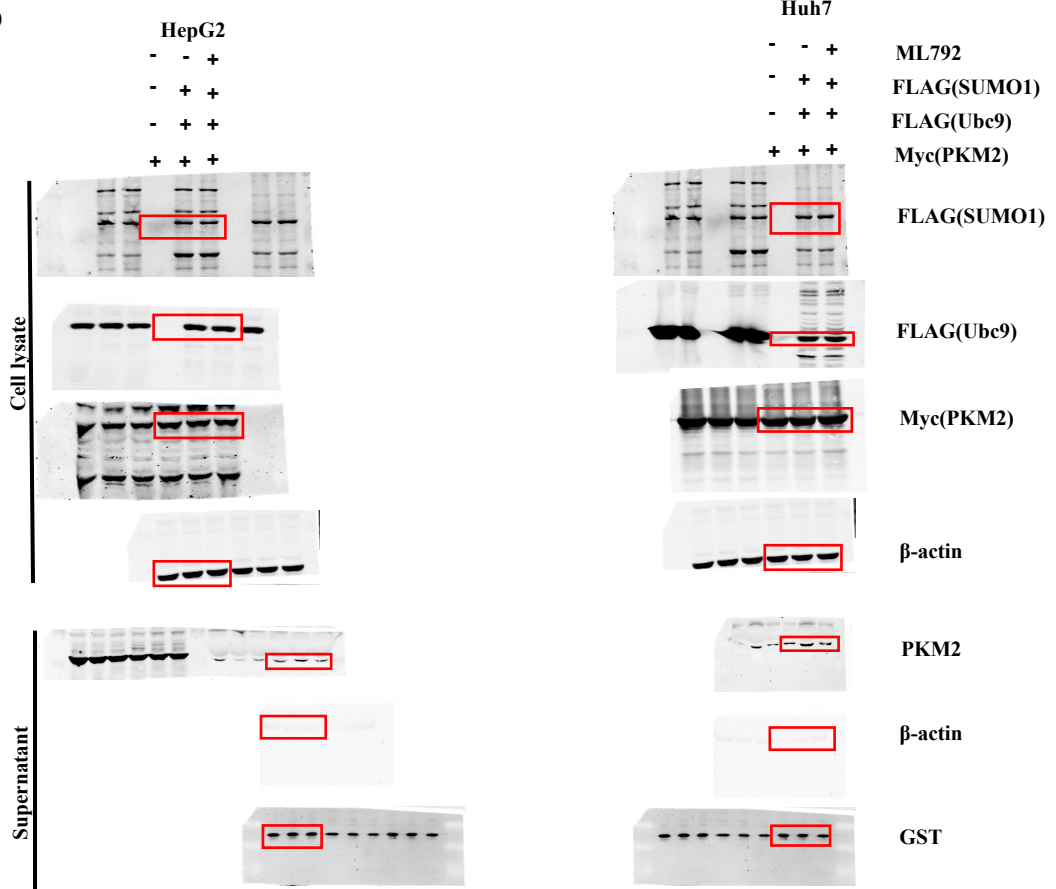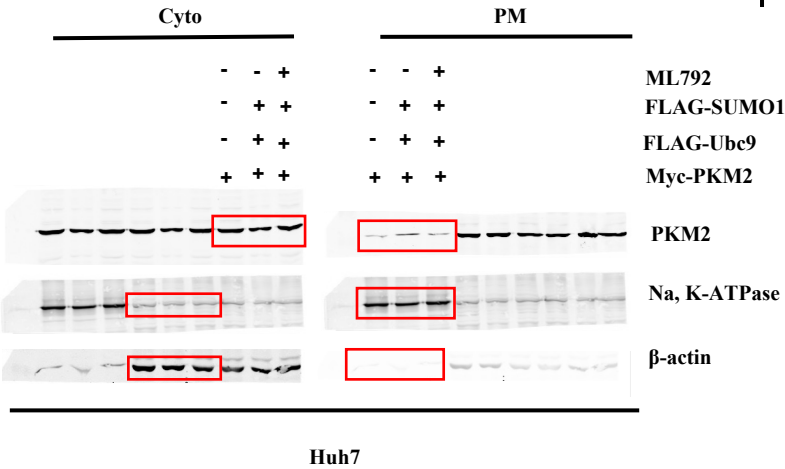

Figure S3

E

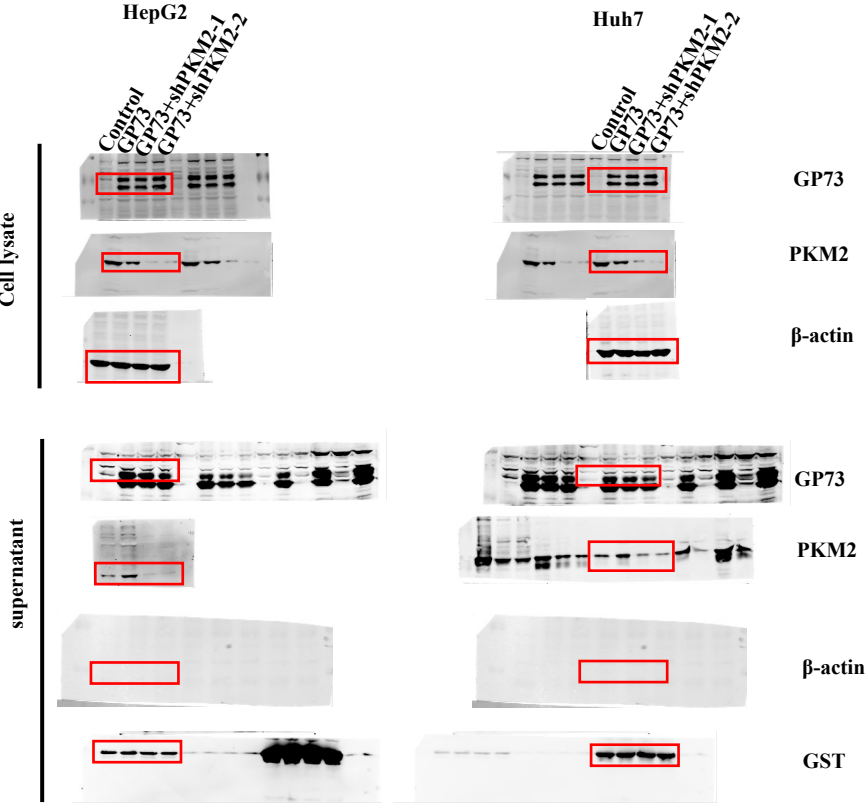

Supplement: Supplementary file 3 — Original western blots [file 41419_2025_7391_MOESM3_ESM.pdf]
